# HOX paralogs selectively convert binding of ubiquitous transcription factors into tissue-specific patterns of enhancer activation

**DOI:** 10.1371/journal.pgen.1009162

**Published:** 2020-12-14

**Authors:** Laure Bridoux, Peyman Zarrineh, Joshua Mallen, Mike Phuycharoen, Victor Latorre, Frank Ladam, Marta Losa, Syed Murtuza Baker, Charles Sagerstrom, Kimberly A. Mace, Magnus Rattray, Nicoletta Bobola

**Affiliations:** 1 School of Medical Sciences, University of Manchester, Manchester, United Kingdom; 2 School of Health Sciences, University of Manchester, Manchester, United Kingdom; 3 Department of Computer Science, University of Manchester, Manchester, United Kingdom; 4 Department of Biochemistry and Molecular Pharmacology, University of Massachusetts Medical School, Worcester, Massachusets, United States of America; 5 School of Biological Sciences, University of Manchester, Manchester, United Kingdom; Columbia University, UNITED STATES

## Abstract

Gene expression programs determine cell fate in embryonic development and their dysregulation results in disease. Transcription factors (TFs) control gene expression by binding to enhancers, but how TFs select and activate their target enhancers is still unclear. HOX TFs share conserved homeodomains with highly similar sequence recognition properties, yet they impart the identity of different animal body parts. To understand how HOX TFs control their specific transcriptional programs *in vivo*, we compared HOXA2 and HOXA3 binding profiles in the mouse embryo. HOXA2 and HOXA3 directly cooperate with TALE TFs and selectively target different subsets of a broad TALE chromatin platform. Binding of HOX and tissue-specific TFs convert low affinity TALE binding into high confidence, tissue-specific binding events, which bear the mark of active enhancers. We propose that HOX paralogs, alone and in combination with tissue-specific TFs, generate tissue-specific transcriptional outputs by modulating the activity of TALE TFs at selected enhancers.

## Author summary

The different anatomical features that develop along the animal body axis are determined by HOX transcription factors. Each member of the HOX family appears in a well-defined territory of the developing embryo, and instruct a specific gene expression program. In stark contrast to their specificity *in vivo*, which is central to developmental programs, HOX transcription factors display highly similar sequence recognition properties *in vitro*. To understand how HOX transcription factors control their specific transcriptional programs *in vivo*, we compared HOXA2 and HOXA3 binding profiles in the mouse embryo. We find that HOXA2 and HOXA3 occupy a large set of high-confidence, non-overlapping genomic regions, that are also bound by three aminoacid loop extension (TALE) transcription factors. We identify three main determinants of HOX paralog-selective binding: recognition of unique variants of the HOX-PBX motif, differential affinity at shared HOX-PBX motifs and, additionally, contribution of tissue-specific transcription factors. These mechanisms result in high-confidence, cooperative HOX-TALE binding at different genomic locations, which bear the mark of active enhancers. We propose that HOX paralogs operate, alone and in concert with tissue-specific transcription factors, to switch on TALE function at selected enhancers.

## Introduction

Gene expression programs instruct and maintain cell fate in embryonic development and adult tissue homeostasis. Transcription factors (TFs) control gene expression by binding to enhancers [1,2]. However, we still have no clear idea of how TFs select their precise sets of target enhancers. While TFs contain DNA binding domains which recognize DNA in a sequence-specific manner, these interactions are typically insufficient to direct a TF to its functional targets.

Transcriptional regulation is mediated by TFs working together, rather than in isolation. The widespread occurrence of collaborative TF binding is imposed by chromatin. A single TF cannot easily compete with nucleosomes to access DNA, but multiple TFs that recognize closely spaced binding sites can effectively displace nucleosomes and indirectly facilitate each other’s binding [3,4]. Such indirect cooperativity can also result in TFs recognizing low affinity sites, i.e. sites that deviate from their optimal consensus *in vitro* [5]. Recent observations indicate that TF cooperativity does not end at binding enhancers: clusters of enhancer-bound TFs concentrate co-activators and other nuclear factors via dynamic fuzzy interactions, driven by their intrinsically disordered regions (IDRs). IDRs function in molecular recognition and mediate the interaction with a diversity of regulatory proteins [6,7] to promote the liquid-liquid phase transition associated with gene activation [8]. Thus, the formation, on DNA segments, of regulatory complexes made of different combinations of factors, is key to activation of gene expression. These distinct combinations of TFs produce virtually inexhaustible flavours of gene expression and cell fate [1].

HOX TFs provide an ideal model to explain how TFs select their target enhancers to direct specific transcriptional programs *in vivo*. They contain a homeodomain (HD), a highly conserved DNA binding moiety shared by hundreds of TFs [9,10]. HD display highly similar sequence recognition properties and bind the same core of four-base-pair sequence TAAT [11], yet HOX TFs function to establish the identity of entirely different body parts along the antero–posterior axis of all bilaterian animals [12,13]. In mammals, there are 39 *Hox* genes, classified into anterior (HOX1-2), central (HOX3–8), and posterior (HOX 9–13) paralog groups [14]. HOX paralogs occupy sequential positions along the chromosome, which are faithfully maintained across evolution [15]. This translates into precise HOX expression codes at different levels of the antero-posterior axis, conferring specific spatial and temporal coordinates to each cell.

HOX association with three amino acid loop extension (TALE) HD TFs PBX, and PBX partner MEIS, is a widely accepted mechanism underlying HOX target specificity [10,16,17]. HOX-TALE cooperativity increases the affinity and sequence selectivity of HOX TFs *in vitro* [16]. *In vivo*, HOXA2 extensively binds with TALE TFs [18] and Ubx and Hth (fly homologs to vertebrate central HOX and MEIS respectively) co-localize in active nuclear microenvironments, suggesting that their interaction may be critical to trigger phase separation [19]. Interestingly, Hox binding selectivity can be observed in the absence of TALE TFs, and is strongly associated with chromatin accessibility [20]. Although the concept of HOX and TALE interaction is long established, we still understand relatively little about the extent and functional significance of HOX-TALE association *in vivo*, where compaction of DNA into chromatin and the distribution of sequence-specific TFs (cell-specific and tissue-specific, but also ubiquitous) can considerably affect TF binding to DNA. Also, how the association with fairly ubiquitous proteins eventually translates into HOX paralog-specific transcriptional outputs *in vivo*, remains unclear.

To understand how HOX TFs execute their specific functions to impart different segmental identity *in vivo*, we compared binding of HOXA2 and HOXA3, an anterior and a central HOX proteins, in the physiological tissues where these TFs are active. Branchial arches (BA) are blocks of embryonic tissues that merge to form the face and the neck in vertebrates. The second and third branchial arch (BA2 and BA3) are the main domains of HOXA2 and HOXA3 expression respectively, and the embryonic areas most affected by inactivation of *Hoxa2* and *Hoxa3* in mouse [21–23]. We find that, in the BAs, HOXA2 and HOXA3 occupy a large set of high-confidence, non-overlapping genomic regions, that are also bound by TALE TFs. We identify three main determinants of HOX paralog-selective binding, resulting in high-confidence cooperative HOX-TALE binding at different genomic locations: recognition of unique variants of the HOX-PBX motif, differential affinity at shared HOX-PBX motifs and, additional contribution of tissue-specific TFs. We propose that HOX paralogs operate, alone and in concert with tissue-specific TFs, to switch on TALE function at selected enhancers.

## Results

### HOXA2 and HOXA3 control diverse processes by targeting different regions of the genome

**Fig 1:**
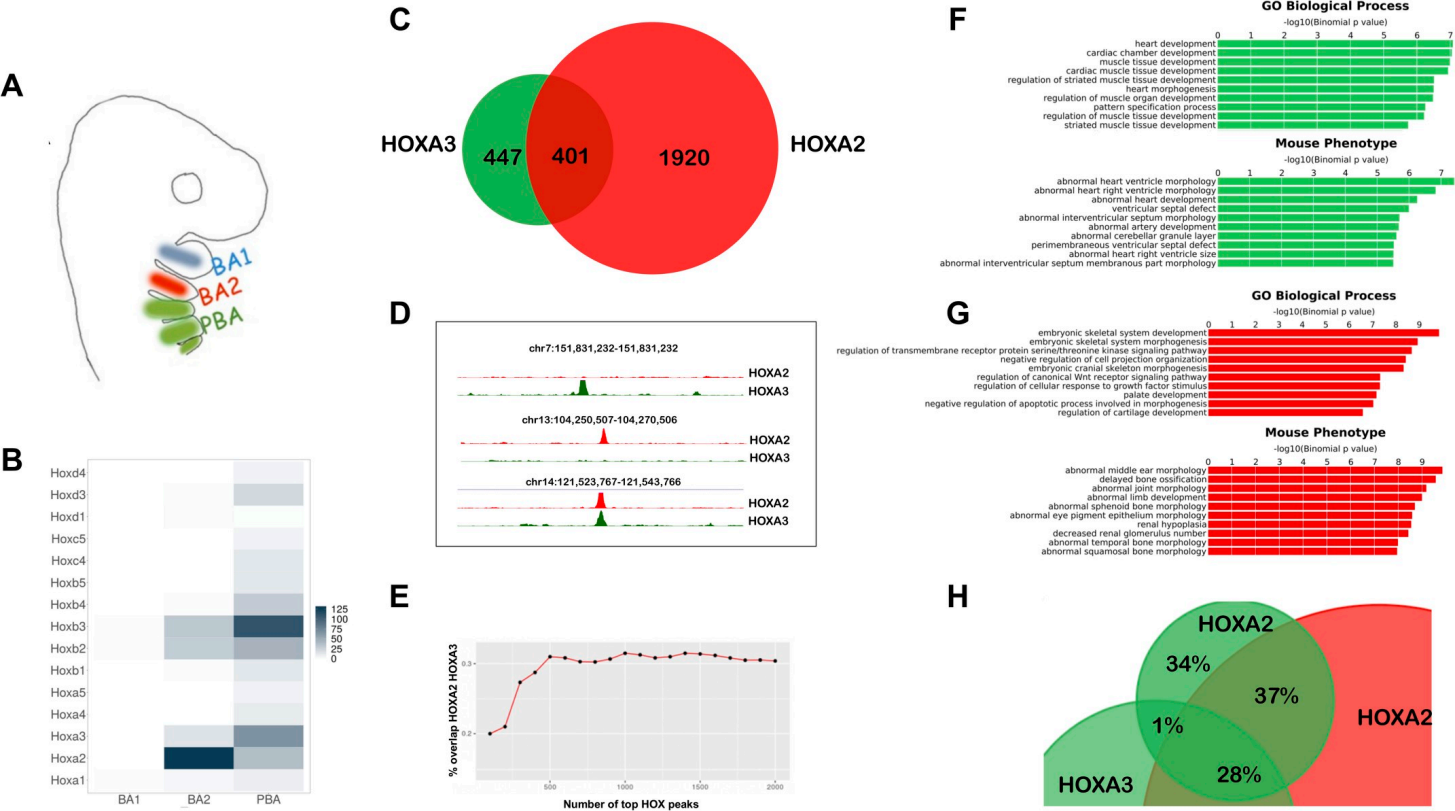
HOXA2 and HOXA3 control diverse processes by targeting different regions of the genome *in vivo*. A. BA organization in mammals. BA3-6 are collectively indicated as PBA. The same colour code (BA2 red, PBA green) is used throughout the manuscript. B. Heatmap of *Hox* expression in E10.5 mouse BA1, BA2 and PBA, based on the normalized expression values count per million (CPM) [30]. C. Overlap of HOXA3 binding in PBA and HOXA2 binding in BA2 (200 nt summits, overlap at least 1 nt). Only peaks with FE≥10 are considered. D. UCSC tracks (mm9) of HOXA3 (green) and HOXA2 (red) specific and shared peaks. E. Overlap (expressed as %) of increasing numbers of top HOXA2 and HOXA3 peaks (ranked by FE). High-confidence peaks show the smallest overlap. FG. GREAT analysis of HOXA3- (F) and HOXA2- (G) specific peaks (non-overlapping, green and red bars respectively) shows association with genes involved in different biological processes and whose mutations generate different phenotypes in mouse. The length of the bars corresponds to the binomial raw (uncorrected) P-values (x-axis values). H. HOXA2 binding in PBA. Overlap of HOXA2 summit regions in PBA (FE ≥10, green) with HOXA2 summit regions in the BA2 (red) and HOXA3 summit regions in the PBA (green); same rule as in C. HOXA2 binding locations are similar in BA2 and PBA.

HOX TFs direct highly specific gene expression programs *in vivo*, but recognize very similar DNA sequences *in vitro*. However, it remains to be determined if HOX specificity of action reflects specificity of binding across the genome *in vivo*, i.e. the binding of paralog HOX TFs to distinct target regions. To establish this, we compared HOXA2 and HOXA3 binding profiles in their physiological domains of expression in the mouse embryo. BAs display an antero-posterior gradient of HOX expression, which replicates *Hox* gene positions on the chromosome (Fig 1A and 1B): BA1 does not express any *Hox* gene, BA2 expresses *Hox2* paralogs, BA3 *Hox3* paralogs, etc. We previously characterized HOXA2 binding in BA2 [18]; here, we profiled HOXA3 binding in BA3-4-6 (hereafter referred to as posterior branchial arches, PBA), the embryonic tissues immediately posterior to the BA2 (identified by the expression of *Hox* paralogs 3–5, Fig 1A and 1B). Using a HOXA3-specific antibody (S1A Fig), we identified 848 peaks with fold enrichment (FE) ≥10, which largely contained a second biological replicate (S1B Fig; S1 Table). TALE TFs (PBX and MEIS) display cooperative binding with HOX and increase HOX binding specificity *in vitro* [16]. De novo motif discovery [24] identified HOX-PBX recognition sequence as the top enriched motif in HOXA3 peaks and uncovered MEIS binding site in the top three sequence motifs (S1C Fig). HOXA3 recognition sites in PBA correspond to HOXA2 motifs in BA2 (S1D and S1E Fig); moreover, the frequency of HOX-PBX motifs is comparable across HOXA2 and HOXA3 peaks (S1F Fig). HOX peaks without canonical HOX-PBX consensus motifs, contain similar (i.e. 1nt mismatch), potential low affinity variants of HOX-PBX sites (S1F Fig). The occurrence of high affinity sites (perfect matches) positively correlates with peak FE, and is highest in top HOXA2 and HOXA3 peaks. Low affinity sites (1 mismatch allowed in TGATNNAT) show the opposite trend and occur with higher frequency in lower confidence binding events (S1G Fig).

We overlapped HOXA2 binding in BA2 (S2 Table) with HOXA3 binding in PBA. About half of HOXA3 peaks are contained in the larger HOXA2 datasets (Fig 1C and 1D). When comparing the same number of peaks for both datasets, ranked by FE, we observed an increasing overlap at lower confidence peaks (Fig 1E), suggesting that HOXA2 and HOXA3 select different sites when binding with higher affinity and are more promiscuous at lower binding levels. Functional association of HOXA3-specific peaks in PBA and HOXA2-specific peaks in BA2 [25] (Fig 1F and 1G) highlights distinct biological processes and mouse phenotypes, including abnormal middle ear, sphenoid, temporal and squamosal bone morphologies, whose morphogenesis is controlled by HOXA2 [21,22]. Remarkably, genes dysregulated in the HOXA2 mutant [26] are significantly represented in the top biological processes and mouse phenotypes associated with HOXA2 peaks (S1H Fig). In contrast HOXA3-specific binding is almost exclusively associated with heart and cardiac muscle development and cardiovascular phenotypes, consistent with the role of HOXA3 in the formation of the main arteries [23,27] (Fig 1F). These observations are in line with HOX functional specificity and indicate that in their physiological domains of expression, HOXA2 and HOXA3 bind in the vicinity of, and potentially control, genes involved in very different processes. *Hoxa2* expression displays a sharp anterior border between BA1 and BA2 and expands in the more posterior PBA (Fig 1A, Fig 4A). We profiled HOXA2 binding in PBA to understand if HOX-specific binding is determined by differences in the BA2 and PBA chromatin environment. We found that HOXA2 peaks in PBA very rarely overlap with HOXA3 ‘only’ peaks in the same tissue (1% overlap), but are largely contained in the pool of HOXA2-specific binding in BA2 and ‘common’ HOXA2 and HOXA3 binding events (Fig 1H). This, combined with the observation that HOXA2 and HOXA3 are largely co-expressed in the same cells in PBA and therefore exposed to the same chromatin environment [28] (see also Fig 4, S1A and S1B Fig) argues against differences in chromatin accessibility being a main determinant of HOX binding. In sum, analysis of HOXA2 and HOXA3 ChIP-seq in their respective domains of expression indicates that different HOX TFs control diverse and specific processes by targeting different regions of the genome *in vivo*. Tissue-specific chromatin accessibility does not appear to be a major determinant in HOX paralogs’ target site selection.

### HOXA2 and HOXA3 select variants of the HOX/PBX motif

**Fig 2:**
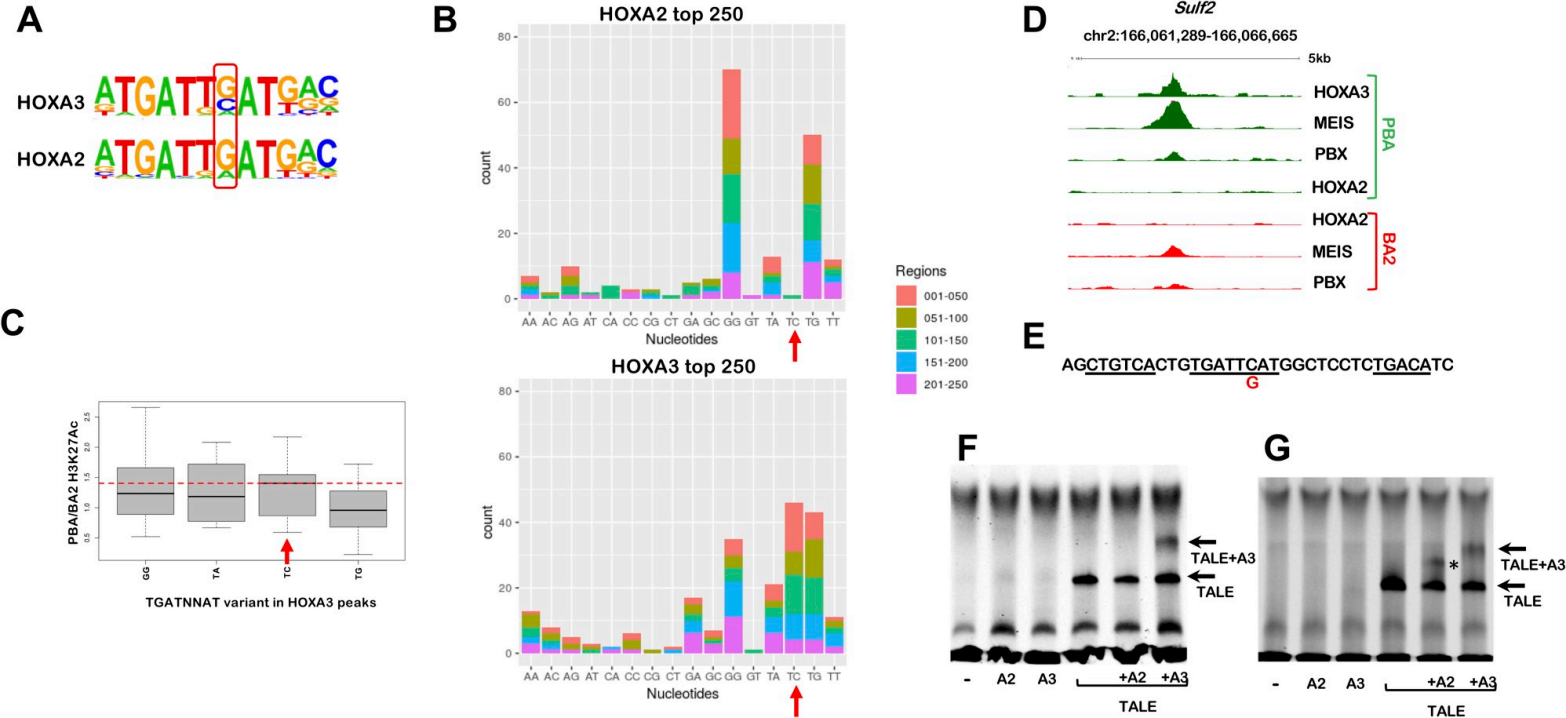
HOXA2 and HOXA3 select variants of the HOX/PBX motif. A. Homer detects different variants of the HOX-PBX motif in top 250 HOXA2 and HOXA3 peaks, with a G/C (HOXA3) or mainly a G (HOXA2) in the second variable position. B. Occurrence of HOX-PBX motif variants (all permutations of the variable nucleotides in TGATNNAT) in top 250 HOXA2 and HOXA3 peaks (ordered into 50 region bins by decreasing FE). The TGATTCAT motif (red arrows) is among the most enriched variants in HOXA3 peaks but does not virtually occur in HOXA2 peaks. C. Distribution of differential H3K27 acetylation (PBA/BA2 ratio) at top 250 HOXA3 peaks containing the four most frequent TGATNNAT variants. Data is ordered based on fold change of acetylation (fold change of normalized read counts) and divided into quartiles. The central quartiles (50% of the data) are shown in the box, while top and bottom whiskers represent the top 25% and bottom 25% (the top and the bottom quartiles) of the data, respectively. HOXA3 peaks containing the TGATTCAT variant are associated with increased enhancer activity in PBA (red line). D. UCSC tracks with HOXA3, HOXA2, PBX and MEIS binding profiles in BA2 (red) and PBA (green) at the *Sulf2* locus, containing TGATTCAT. No HOXA2 binding is detected in BA2 or PBA. E. Sequence of HOXA3 peak summit in D, corresponding to the probe used in F. The TGATTCAT motif (underlined) is flanked by two MEIS motifs (also underlined); the C➔G substitution tested in G is indicated in red. F. HOXA3 can selectively bind the *Sulf2* probe in complex with PBX/MEIS. Incubation of the *Sulf2* probe with TNT reticulocytes expressing HOXA2, HOXA3, MEIS/PBX, HOXA2/MEIS/PBX or HOXA3/MEIS/PBX. MEIS/PBX bind the *Sulf2* probe in combination (arrow). Addition of HOXA3 to the probe results in the formation of a complex only in the presence of PBX/MEIS (arrow). No complex is formed when PBX/MEIS are co-translated with HOXA2. G. Same experiment as in F, using a mutant *Sulf2* probe (the nucleotide substitution is shown in E). HOXA2 can bind the mutant probe in combination with MEIS/PBX (asterisk), similar to HOXA3 (arrow).

The observations above indicate that HOXA2 and HOXA3 select different genomic sites *in vivo*, while at a first glance, they recognize very similar DNA sequences. To investigate the determinants of HOX binding specificity, we focused on high confidence HOXA2 and HOXA3 peaks (top 250, ranked by FE), which display the lowest overlap across the genome (Fig 1E). As expected, both HOXA2 and HOXA3 top peaks were enriched in the HOX/PBX motif (present in 66.4% and 68.4% of HOXA2 and HOXA3 respectively). *De novo* motif discovery identified enrichment of a HOX-PBX variant in HOXA3 top 250 peaks, which contains a C in the second variable position (i.e. TGAT**NC**AT) (Fig 2A). We next counted the distribution of all permutations of the TGATNNAT motif in top HOXA2 and HOXA3 peaks and found the TGAT**TC**AT variant to be highly differentially enriched in HOXA3 peaks (Fig 2B). This sequence, which is highly represented in HOXA3 top peaks (~ 20%), is almost excluded from HOXA2 peaks (Fig 2B). Supporting functional significance, HOXA3 peaks containing TGAT**TC**AT display increased acetylation levels (a mark of active enhancers) [29] in HOXA3-expressing tissues (Fig 2C). In addition, while HOXA2 peaks display a very high representation of TGAT**GG**AT and TGAT**TG**AT, HOXA3 high confidence binding allows higher variability (four variants are counted > 20 times in HOXA3 peaks as opposed to only two variants in top HOXA2 peaks) (Fig 2B). The highest differential enrichment of TGATNNAT variants is observed in top HOXA2 and HOXA3 peaks (S2A Fig), which also display minimal overlap across the genome (Fig 1E); this suggests that the ability to recognize different sequences plays a role in genomic site selections. Finally, the majority of HOXA3 (158/250) and HOXA2 (160/250) top peaks contain MEIS recognition motif, at a preferential distance of less than 20 nt from the TGATNNAT motif (S2B Fig). The *Sulf2* locus exemplifies HOXA3 specific binding in PBA: HOXA3 peak summit contains a single TGAT**TC**AT motif and displays high HOXA3 occupancy, but no detectable HOXA2 binding (Fig 2D and 2E). We used electrophoretic mobility shift assay (EMSA) to establish if HOXA3 preferentially recognizes the TGATTCAT sequence *in vitro*. We did not observe any HOXA2 or HOXA3 binding to the *Sulf2* probe (Fig 2F). Incubation with PBX and MEIS resulted in a probe shift. Addition of HOXA3, but not HOXA2, resulted in the formation of a ternary complex, indicating that HOXA3 can bind this site in combination with PBX and MEIS, while HOXA2 cannot (Fig 2F). In support of this conclusion, converting TGATT**C**AT to TGATT**G**AT (a single nucleotide substitution in the *Sulf2* probe), enables binding of HOXA2, in addition to HOXA3 (Fig 2G, S2C Fig). Finally, we observed no synergy between HOXA3, PBX and MEIS on the *Sulf2* enhancer in transfection assays (S2D Fig), suggesting that additional TFs could function with HOXA3 at this site; of note this HOXA3-specific site closely resembles AP-1 TF consensus sequence [24]. These results indicate that HOXA3 and HOXA2 have diverse binding preferences and uncover the existence of sites that are exclusively recognized by HOXA3.

### HOXA2 molecular control of BA2 identity

**Fig 3:**
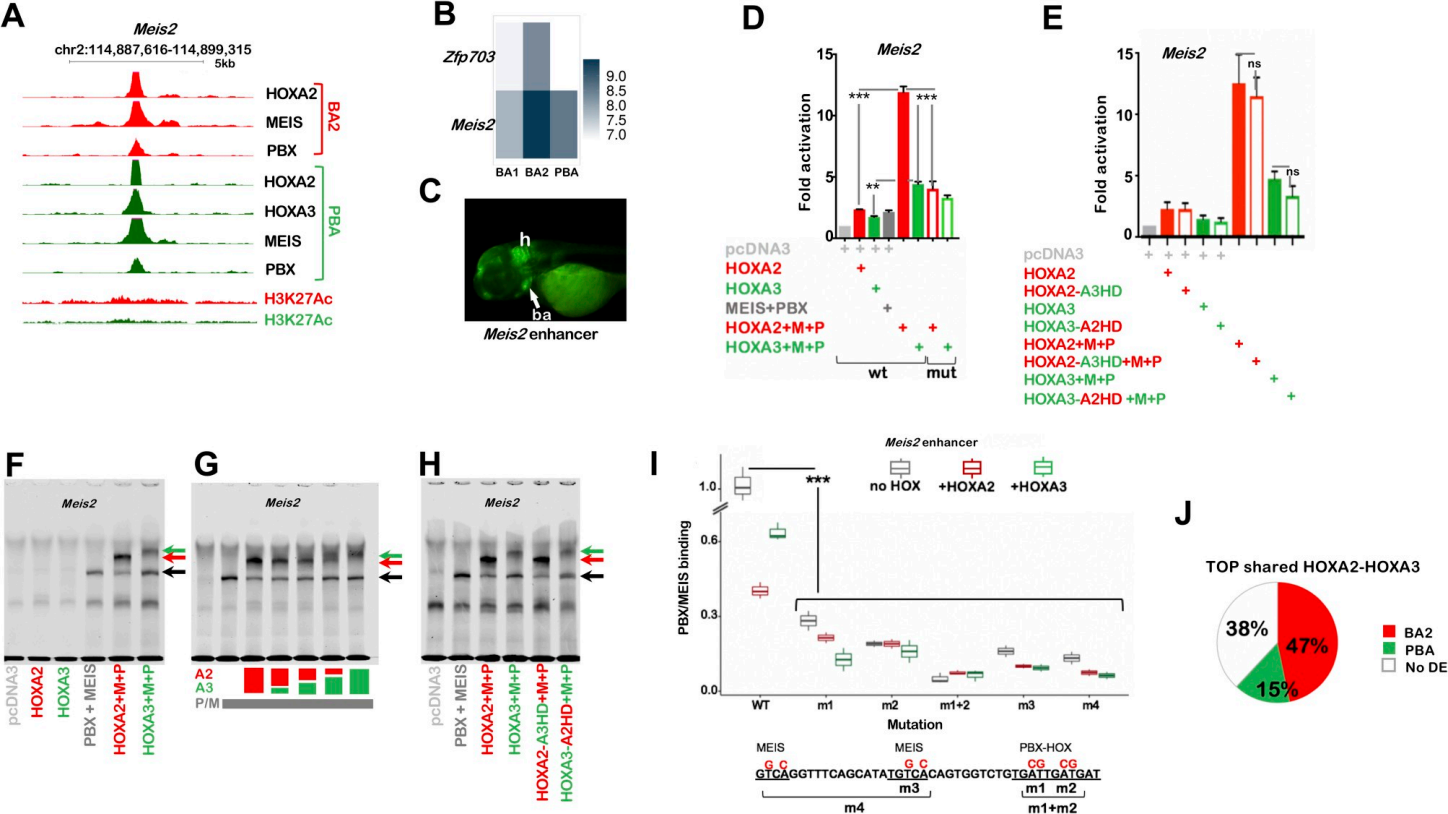
HOXA2 control of target enhancers. A. UCSC tracks of HOXA2, HOXA3, PBX, MEIS binding and H3K27 acetylation profiles in BA2 (red) and PBA (green) at the *Meis2* locus. Strong HOX and TALE binding is observed in both tissues, with higher acetylation levels in BA2. B. Heatmap shows *Meis2 and Zfp703* expression in E11.5 mouse BA1, BA2 and PBA, based on the normalized expression values CPM [30]. C. *Meis2* enhancer is active in the hindbrain (h) and the BAs (ba, arrow) of developing zebrafish (72 hours post fertilization), which correspond to *Meis2* expression domains in mouse [18]. The enhancer sequence spans the 200nt summit of HOXA2 peak in A. D. Luciferase activity driven by *Meis2* enhancer co-transfected with *Hoxa2* (red bar) or *Hoxa3* (green bar) in combination with *Meis2* and *Pbx1a* expression vectors in NIH3T3 cells. The combination of *Hoxa2* with *Meis2* and *Pbx1a* results in the highest activation. Changing the HOX-PBX site (empty bars, mutant sequence in F) reduces HOX-TALE activation. E. Luciferase activity driven by *Meis2* enhancer co-transfected with *Hoxa2-a3HD* (red empty bar) or *Hoxa3-a2HD* (green empty bar) and *Meis2* and *Pbx1a*. Values shown in DE represent fold activation over basal enhancer activity and are presented as the average of at least two independent experiments, each performed in triplicate. Error bars represent the standard error of the mean (SEM). One-way ANOVA with post-hoc Tukey HSD (Honestly Significant Difference) test: ** = p<0.005; *** = p<0.0005. F-G. Incubation of the *Meis2* probe with TNT reticulocyte expressing HOXA2, HOXA3, MEIS/PBX, HOXA2/MEIS/PBX or HOXA3/MEIS/PBX as indicated. F. MEIS and PBX bind the *Meis2* probe together (black arrow, see also S3E and S3F Fig). Addition of HOXA2 and HOXA3 results in a trimeric protein complex (red and green arrows respectively). Co-translation of HOXA2 with PBX/MEIS gives rise to a stronger trimeric complex relative to HOXA3 and, accordingly, to a decrease in PBX-MEIS binding as a dimer (black arrow; see also S3G Fig for quantification). G. HOX binding to *Meis2* probe, alone and in combination. The total HOX dose is unvaried and corresponds to 100%; HOXA2 and HOXA3 are translated alone and co-translated with each other at different ratios, as follows: 100% HOXA2; 75% HOXA2 + 25% HOXA3; 50% HOXA2 + 50% HOXA3; 25% HOXA2 + 75% HOXA3; 100% HOXA3. Colour coded % are indicated below the gel; HOXA2 is red and HOXA3 is green. HOXA2 forms a stronger trimeric complex (red arrow) than HOXA3 (green arrow) at comparable concentrations. PBX/MEIS dimeric complex, black arrow (see also S3H and S3I Fig for quantification). H. Swapping HOXA3-HD with HOXA2-HD does not improve the ability of HOXA3 to form a ternary complex with PBX and MEIS (green arrows), and does not decrease HOXA2 binding with MEIS and PBX (red arrows). Adding HOXA2 (or HOXA2-A3HD) results in higher intensity of the trimeric complex (red arrows) and lower intensity of TALE dimeric complex relative to HOXA3 (or HOXA3-A2HD), as observed in F. I. Quantification of MEIS/PBX binding to *Meis2* mutant probes. The sequence of *Meis2* wild-type and mutant probes is shown below: HOX-PBX and MEIS motifs are underlined and nucleotide substitutions are shown in red. Mutants are as follows: m1 and m2 contain mutations in each half of the HOX-PBX site, m1+m2 in both halves; m3 contains mutations in MEIS site closest to HOX-PBX motif and m4 bears mutations in both MEIS sites. MEIS/PBX binding to each mutant probe is expressed as relative percentage to PBX/MEIS binding to *Meis2* wild-type in the absence of HOX. Binding of PBX/MEIS dimer was quantified in the absence (black boxes) and in the presence of HOXA2 (red boxes) and HOXA3 (green boxes). Values are the result of three independent experiments; for accurate quantification, each gel was run with the reference condition (PBX/MEIS bound to *Meis2* wild-type in the absence of HOX). All mutations significantly decrease PBX/MEIS binding (p<0.0005, one-way ANOVA with post-hoc Tukey HSD test). J. Top HOXA2 and HOXA3 overlapping peaks (total of 60/250 intersecting HOXA2 and HOXA3 top peaks) are more frequently associated with genes with higher expression in BA2 (red) relative to PBA (green). The white portion of the pie chart refers to genes that are not differentially expressed between BA2 and PBA (no DE). Gene association is based on GREAT standard association rules; expression levels are extracted from E11.5 RNA-seq [30].

In contrast to HOXA3, which displays unique binding preferences for TGATTCAT, we did not detect HOX-PBX variants exclusively recognized by HOXA2. To investigate the mechanisms underlying HOXA2 control of BA2 identity, we examined HOXA2 binding events (top peaks) in the vicinity of well-established HOXA2 downstream targets. *Meis2* and *Zfp703* are associated with high levels of HOXA2 binding [18] (Fig 3A and S3A Fig) and are downregulated in *Hoxa2* null BA2 [26]. In addition, consistent with *Meis2* and *Zfp703* expression being HOXA2-dependent, they are expressed at higher levels in BA2 than the HOX-less BA1 and the HOXA3-positive PBA (Fig 3B) [18,30]. *Meis2* and *Zfp703* loci exhibit high HOXA2 and HOXA3 binding in their vicinity, suggesting their associated chromatin is largely accessible in both BA2 and PBA (Fig 3A and S3A Fig). The sequence contained in *Meis2* peak is active in the main domains of HOXA2 expression, the hindbrain and BAs in zebrafish (Fig 3C). When tested in luciferase assays in NIH3T3 cells, the *Meis2* functional enhancer displays higher activity in the presence of HOXA2, in combination with MEIS and PBX, relative to HOXA3 (Fig 3D). *Meis2* enhancer activity is largely dependent on the integrity of its HOX-PBX site (Fig 3D). Adding TALE to the *Zfp703* putative enhancer also significantly increased the difference in HOXA2 and HOXA3 activation abilities (S3B Fig). However, adding HOXA2 and HOXA3 alone resulted in higher activation of the *Zfp703* putative enhancer relative to *Meis2* enhancer (presumably due to the presence of a TAAT site within the HOX/PBX motif) and including TALE did not significantly change HOXA2 activation. Similar to the *Meis2* enhancer, disruption of the HOX/PBX site significantly decreased luciferase activity (S3B and S3C Fig). Finally, HOXD3, another representative of HOX paralog group 3, whose members have largely overlapping functions *in vivo* [27], also displayed a lower activating capacity than HOXA2 on *Meis2* enhancer (S3D Fig). In sum, HOXA2 is more efficient at activating both target regions, in the presence of PBX and MEIS. To understand if this reflects HOXA2 and HOXA3 different DNA binding properties, we generated HOX chimeric proteins by swapping HOXA2 and HOXA3 DNA-binding HDs. We found that providing HOXA2 with HOXA3 HD did not substantially change the ability of HOXA2 to activate transcription from the *Meis2* enhancer (Fig 3E). Similarly, the ability of HOXA3 to transactivate the *Meis2* and *Zfp703* enhancers, alone or in complex with MEIS and PBX, was not improved by swapping HOXA3 HD with HOXA2 HD (Fig 3E and S3B Fig). As HOX TFs cooperate with MEIS and PBX to activate target enhancers and activation relies on the presence of an intact HOX/PBX recognition motif, HOXA2 and HOXA3 diverse activation properties may depend on their respective abilities to interact with PBX and MEIS on DNA. On their own, HOXA2 and HOXA3 weakly bind the *Meis2* enhancer, but interact with PBX and MEIS to form a ternary protein complex on DNA (Fig 3F, see also S3E and S3F Fig). A larger fraction of MEIS-PBX complex is bound by HOXA2, while addition of HOXA3 results in a less robust super-shift of the dimeric band (Fig 3F and S3G Fig). To confirm these observations, we co-translated HOXA2 and HOXA3 at different ratios and found that, at all concentrations tested, HOXA2 forms a stronger trimeric complex on the *Meis2* probe, relative to HOXA3 at an equivalent concentration (Fig 3G and S3H Fig). Concomitantly, HOXA2 shifts more of the dimeric band (PBX/MEIS) into a trimeric complex (Fig 3G and S3I Fig). We did not detect any slower complexes, suggesting that this DNA sequence can only bind HOXA2 or HOXA3 at one time (Fig 3G and S3J Fig). We observed the same binding patterns using HOX chimeras: swapping HOXA3-HD with HOXA2-HD did not improve the ability of HOXA3 to form a ternary complex with PBX and MEIS, and did not affect HOXA2 ability to bind DNA in complex with MEIS and PBX (Fig 3H, S3G Fig). Next, we investigated the contribution of HOX-PBX and MEIS recognition motifs to HOX and TALE binding to DNA. We systematically introduced mutations in individual sites and tested the resulting probes in EMSA. All mutations strongly reduced the formation of PBX-MEIS dimers (Fig 3I and S3K Fig). We only detected formation of HOX-MEIS-PBX trimeric complexes when one of the two MEIS recognition motifs was still intact, and even in this case complex formation was strongly decreased (S3K Fig), highlighting an essential role of MEIS for HOX binding to DNA. Together, these results indicate that the differential ability of HOXA2 and HOXA3 to bind DNA and activate transcription does not depend on HOX-DNA binary binding. Rather, it reflects differential abilities to form functional HOX-TALE complexes on DNA and is encoded by residues outside the HOXA2 and HOXA3 HD. In summary, while HOXA2 does not exclusively access its sites (HOXA3 can bind as well, Fig 3A), HOXA2 binds more efficiently with TALE at these sites, leading to increased transcriptional activation. Consistently, shared high-confidence HOXA2 and HOXA3 binding events are largely associated with genes expressed at higher levels in the BA2 (Fig 3J). Thus, at least in part, HOXA2 instructs the formation of a BA2 by raising the expression levels of HOX-regulated genes. Crucially, among these genes is *Meis2*, which encodes a critical component for BA2 identity [18].

### HOXA2 activity is decreased in PBA

**Fig 4:**
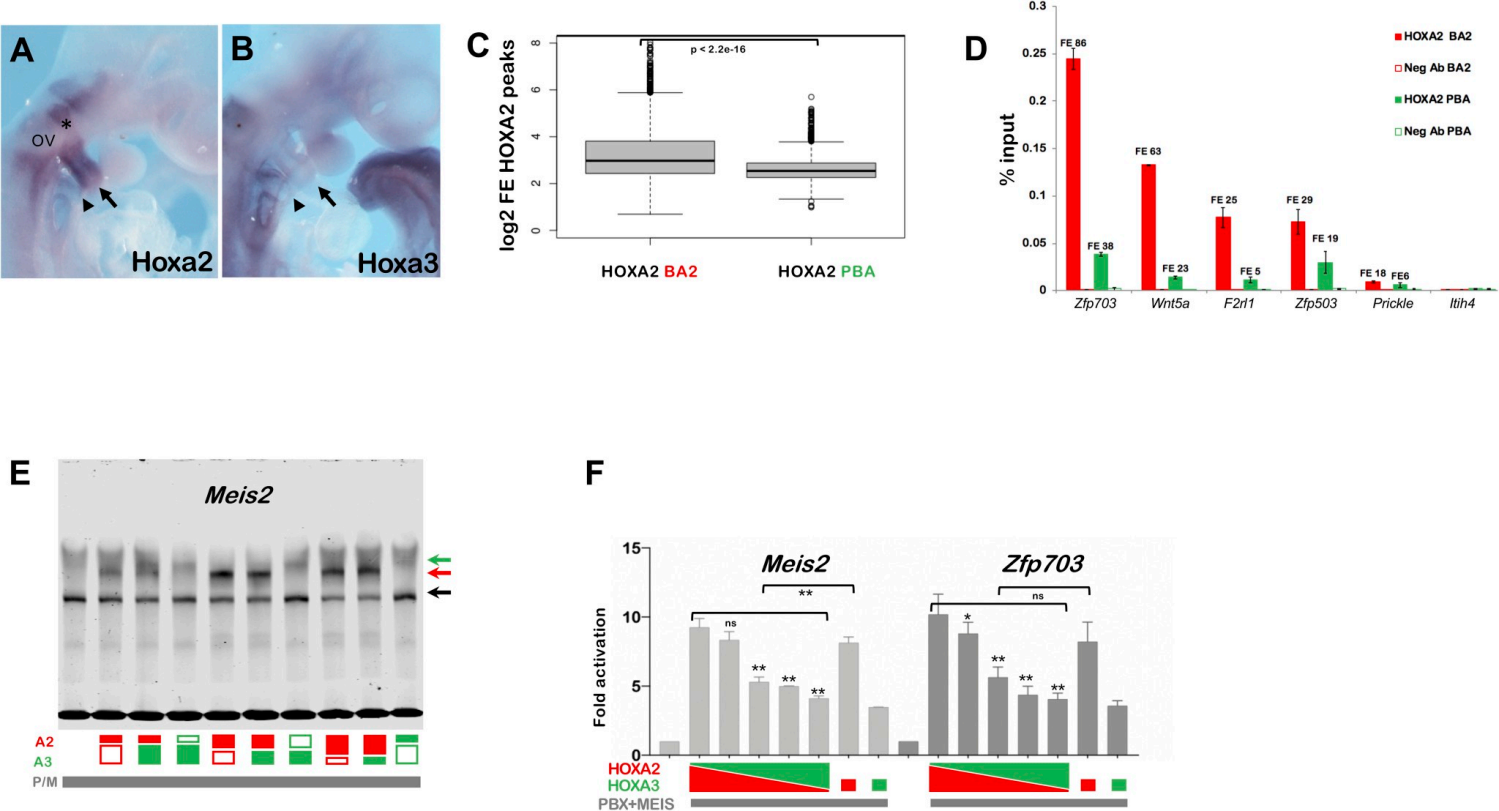
AB. *In situ* hybridization on E9.5 embryos, using *Hoxa2* (A) and *Hoxa3* (B) probes. A. *Hoxa2* is highly expressed in the neural crest migrating from rhombomere 4 (asterisk) to the BA2 (arrow). The portion of neural crest migrating just below the otic vesicle (OV) into the BA3 (arrowhead) is also *Hoxa2*-positive. B. *Hoxa3* is expressed in the BA3 (arrowhead). C. Distribution of HOXA2 peaks FE in BA2 and PBA. HOXA2 peaks in BA2 and PBA are ordered based on log2 FE and divided into quartiles. The central quartiles (50% of the data) are shown in the box, while top and bottom whiskers represent the top 25% and bottom 25% (the top and the bottom quartiles) of the data, respectively. HOXA2 peaks display a significantly higher FE in BA2 compared to PBA (p < 2.2e-16; one sided Welsh two sample t-test). D. Comparison of HOXA2 binding in BA2 (red bars) and PBA (green bars) by ChIP-qPCR. Enrichment of each region following immunoprecipitation with HOXA2 and IgG negative control antibody (Neg Ab) is calculated as percentage input; numbers indicate the corresponding FE values in HOXA2 ChIP-seq (BA2 and PBA). Peaks are labelled by their closest genes. *Itih4* is a negative control (unbound region). Values represent the average of duplicate samples, and error bars indicate the SEM. E. HOX binding to *Meis2* probe, alone and in combination. Co-translation of HOXA2 with PBX/MEIS and with or without HOXA3; the same applies to HOXA3. The highest HOX dose corresponds to 100%; HOXA2 (red) is translated at progressively decreasing levels, either alone, or co-translated with progressively increasing levels of HOXA3 (green) (e.g. 75% HOXA2; 75% HOXA2 + 25% HOXA3; 50% HOXA2; 50%HOXA2 + 50% HOXA3; 50% HOXA3 as indicated below the gel). At 50% and 25% levels, HOXA2 forms a significantly stronger trimeric complex (red arrow) on its own than when HOXA3 is added. PBX/MEIS dimeric complex, black arrow; HOXA3/PBX/MEIS trimeric, green arrow (see also S4C Fig). F. Luciferase activity driven by *Meis2* and *Zfp703* enhancers co-transfected with expression vector for *Hoxa2* or *Hoxa3*, alone, or at diverse ratio of *Hoxa2* to *Hoxa3* (3:1; 2:2; 1:3) as indicated. All samples contain *Meis2* and *Pbx1a* expression vectors; *Hoxa2* and *Hoxa3* are added as indicated. For both enhancers, luciferase activity decreases as *Hoxa2* is progressively replaced by *Hoxa3* (asterisks indicate significant difference relative to full dose of *Hoxa2*); for *Meis2*, enhancer activation is significantly higher with half dose of *Hoxa2* alone, relative to *Hoxa2* and *Hoxa3* together. Values represent fold activation over basal enhancer activity and are presented as the average of at least two independent experiments, each performed in triplicate. Error bars represent the SEM. One-way ANOVA with post-hoc Tukey HSD test: * = p<0.01; ** = p<0.001.

The above results show that, on its target enhancers, HOXA2 functions more efficiently with TALE relative to HOXA3. Given that HOXA2 is expressed in both the BA2 and in the PBA, why does HOXA2 not instruct a BA2-specific program in the PBA as well? More posterior *Hox* genes are typically able to repress the expression (and suppress the function) of more anterior genes, a process termed ‘posterior prevalence’ [15]. Indeed, *Hoxa2* highest expression is detected in the BA2, while *Hoxa2* is expressed at lower levels in *Hoxa3* main domain of expression, the BA3 (Fig 4A and 4B and Fig 1B). A lower nuclear concentration of HOXA2 is expected to reduce HOXA2 binding to DNA (Fig 3G and S3H Fig). However, to assess how changes in HOXA2 dose affect binding genome-wide, we compared HOXA2 binding in BA2 and in PBA. While HOXA2 binds similar locations in BA2 and PBA (Fig 1H), HOXA2 binding levels are significantly higher in BA2 (Fig 4C, see also S3A Fig). This is further confirmed by quantitative analysis of selected immunoprecipitated regions (Fig 4D). Relative to the BA2, the PBA display lower levels of HOXA2 and also express HOXA3 (Fig 1B). Indeed single cell analysis of E10.5 post-otic cranial neural crest, which includes the PBA, [28] (S4A and S4B Fig) confirms that HOXA2 and HOXA3 are largely expressed in the same cells. Therefore, we investigated the effect of decreasing HOXA2 levels while increasing HOXA3 levels on HOXA2 target enhancers. Using EMSA, we found that at lower HOXA2 levels, the addition of HOXA3 significantly reduced binding of HOXA2 to the *Meis2* probe, together with PBX/MEIS (Fig 4E, S4C Fig). Similarly co-expressing HOXA2 and HOXA3 in NIH3T3 cells reduced activation of HOXA2 target enhancers *in vitro* (Fig 4F); for *Meis2*, halving the amount of HOXA2 (50% HOXA2) resulted in higher activation compared to doubling the levels of HOX proteins (50% HOXA2 + 50% HOXA3). While these observations are entirely consistent with the observed lower levels of HOXA2 targets in PBA (*Meis2*, *Zfp703*), it is unknown if expression of these genes in the PBA is still dependent on HOXA2 or HOX paralog 3. Provided expression of *Meis2*, *Zfp703* is required for correct PBA development, as this area does not display any phenotype in the absence of HOXA2 [21,22], either HOXA2 is not required for their expression, or its function can be fully compensated by HOX paralog 3. In conclusion, both a lower dose of HOXA2 and the presence of HOXA3 (possibly other paralog 3 as well) decrease HOXA2 binding and activating abilities in the PBA. This effect, combined with the lower efficiency of HOXA3 to activate HOXA2 targets, dampens HOXA2 transcriptional program in the PBA.

### HOX cooperates with MEIS

**Fig 5:**
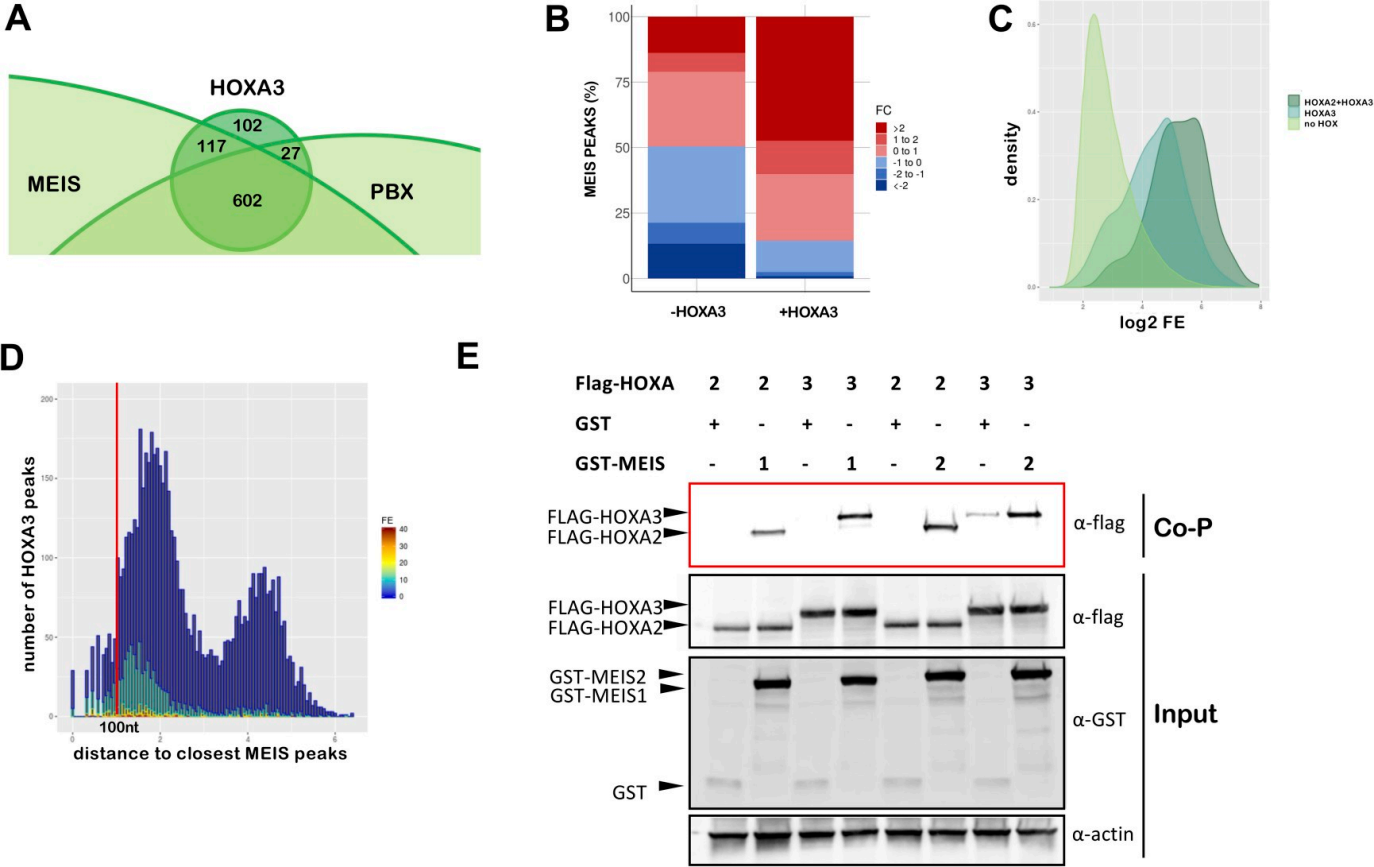
HOX cooperate with MEIS. A. Overlap of HOXA3 with MEIS and PBX peaks in the same tissue (PBA) and at the same embryonic stage (E11.5) (200nt summit regions, overlap at least 1nt). The proportional Venn diagram is cropped to focus on HOXA3 peaks. B. Barplots of fold change in MEIS binding levels in PBA versus BA1. Regions co-occupied by MEIS with HOXA3 in PBA generally display higher MEIS binding levels in PBA (HOX-positive) relative to the HOX-negative BA1. In contrast, MEIS binding not overlapping HOXA3 can be higher in BA1 or in PBA. Fold changes in MEIS peaks were calculated using EdgeR (see also S7 Fig). C. Distribution of FE across different groups of MEIS peaks (PBA). MEIS peaks are sorted into: peaks not overlapping HOX (light green), MEIS peaks overlapping HOXA3 only (‘exclusive’ peaks, i.e. not overlapping HOXA2 in PBA, darker green) and MEIS peaks overlapping HOXA2 and HOXA3 (darkest green). Kernel density plot shows that MEIS peaks overlapping HOX display higher FE relative to MEIS only peaks. D. Distance of HOXA3 peaks relative to MEIS peaks (PBA). HOXA3 peaks are binned according to their log_10_ distance to the nearest MEIS peak and labelled according to FE (high FE, dark red bars; low FE, dark blue bars). High HOXA3 peaks mainly occur within 1kb of a MEIS peak. E. Co-precipitation assays. HEK293 cells were co-transfected with expression vectors for FLAG-tagged HOXA2 or HOXA3 and GST-tagged MEIS1, GST-tagged MEIS2 or GST alone. Protein interactions were assayed by co-precipitation on glutathione beads directed toward the GST tag and eluted proteins analysed by western blotting to detect the presence of FLAG-HOXA2 or FLAG-HOXA3 (red box, Co-P). Cell lysates were analysed by western blotting prior to co-precipitation to detect protein expression (input).

Our results indicate that HOX selectivity is displayed in concert with TALE. Generally, binding with TALE appears to be a dominant feature of HOX binding in the BAs. HOX peaks are enriched in HOX-PBX and MEIS motifs and similar to HOXA2 in BA2 [18], HOXA3 peaks overlap almost entirely with MEIS and PBX peaks in the same embryonic tissue at the same stage (Fig 5A, S5A Fig). We previously discovered that HOXA2 switches its transcriptional program by increasing binding of MEIS TFs to potentially lower-affinity sites across the genome [18]. We investigated if HOXA3 can similarly increase MEIS binding levels. The fraction of MEIS peaks that overlaps HOXA3 binding displays higher FE in PBA, relative to the HOX-free BA1 (Fig 5B). *Hoxa2* is also expressed in PBA, where it could be entirely responsible for the observed increase in MEIS binding. Therefore, to assess HOXA3 unique contribution to MEIS binding increase, we extracted HOXA3-specific binding. We found that MEIS peaks in PBA that overlap HOXA3 ‘exclusive’ peaks, display higher FE (relative to MEIS non-overlapping HOX), indicating that HOXA3 also increases binding of MEIS (Fig 5C), similar to HOXA2 in BA2 [18]. Reciprocally, co-occupancy with MEIS enhances HOXA3 binding (Fig 5D). Both HOXA2 and HOXA3 interact with MEIS1 and MEIS2 in co-precipitation experiments (Fig 5E). Cooperativity, an inherent feature of TF binding to DNA, can arise by protein-protein interactions (direct cooperativity) or by collective binding of TFs to nearby sequences and nucleosome displacement (indirect cooperativity) [31]. The observations that HOX and MEIS display higher binding levels, when binding chromatin together, and interact to form a complex, identify direct cooperativity as the mechanism underlying HOX-TALE binding to chromatin. Cooperativity with MEIS appears to be a general operational principle of HOX TFs as, similar to HOXA2 and HOXA3, MEIS co-occupancy with HOXA1 and HOXA9 is associated with the highest MEIS binding levels in mouse embryonic stem cells [32] and bone marrow cells [33] respectively (S5B–S5D Fig). In sum, HOX directly cooperate with TALE on chromatin. HOXA2 and HOXA3 diverse sequence preferences and binding affinities predict that HOX paralogs preferentially cooperate with distinct subsets of TALE binding events.

### MEIS ‘ubiquitous’ binding is converted into tissue-specific enhancer activity

**Fig 6:**
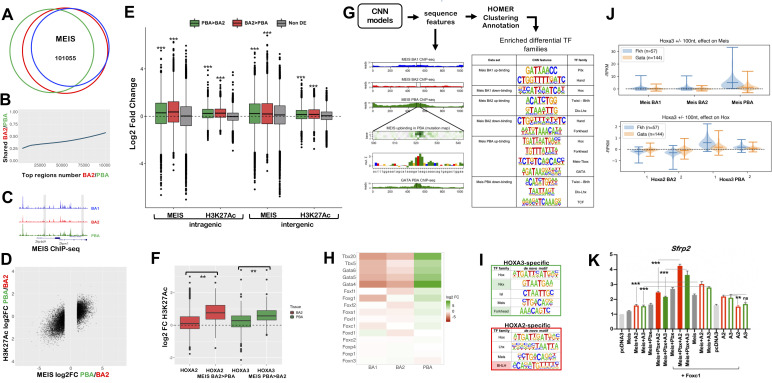
A. Proportional Venn diagram shows highly overlapping binding of MEIS in BA1, BA2 and PBA. MEIS peaks were combined and re-centered using DiffBind; out of 215830 MEIS peaks, 101055 are in common between the three tissues. B. Top MEIS peaks are different in BA2 and PBA. At high FE, there is a lower overlap between MEIS binding in BA2 and PBA. The ratio of MEIS peaks, which are common to BA2 and PBA, increases as FE decreases. C. UCSC tracks illustrate the different MEIS binding levels in BA1-BA2-PBA at the *Zfp496* and *Zfpm*1 loci. Instances of common MEIS peaks higher in one tissue (PBA) are shaded. D. Correlation plot of differential MEIS binding and differential acetylation (enhancer activity) at intergenic regions (PBA versus BA2). Each point corresponds to a region with MEIS log_2_ fold change >1 (FC>2); the corresponding H3K27ac value is plotted. Changes in MEIS binding levels are positively correlated with increased enhancer activity in the same tissue (correlation = 0.73). E. Boxplots of MEIS binding and H3K27Ac levels (log_2_ fold change) associated with differentially expressed genes. Peaks are associated with intragenic and intergenic (+/-100kb from transcription start and end sites) regions of differentially expressed genes (higher expression in PBA, green; higher expression in BA2, red; non-differentially expressed, grey). Irrespective of location, MEIS binding and acetylation levels are significantly higher (p<2.2e-16, one-sided t-test) when associated with genes with higher expression in the same tissue, relative to MEIS binding and acetylation associated with genes expressed at the same levels in BA2 and PBA. Data is ordered based on fold change of acetylation and MEIS binding (fold change of normalized read counts) and divided into quartiles. The central quartiles (50% of the data) are shown in the box, while top and bottom whiskers represent the top 25% and bottom 25% of the data, respectively. F. Changes in H3K27ac (log_2_RPKM) in BA2 and PBA for all HOX peaks and for HOX peaks overlapping MEIS differential binding higher in BA2 (HOXA2 peaks) and higher in PBA (HOXA3 peaks). Data is ordered based on fold change of acetylation (fold change of normalized read counts), as described above. HOX binding generally increases H3K27Ac; peaks associated with increased MEIS binding display a significantly higher increment of H3K27Ac in the same tissue (One-way ANOVA with post-hoc Tukey HSD; ** = p<0.001). G. CNN models of MEIS differential peaks uncover enrichment of tissue-specific sequence motifs as described [35]. MEIS binding was classified in six categories (i.e. peaks with higher/lower binding in BA1, BA2, PBA). CNN analysis identifies tissue-specific sequence features in each class of MEIS peaks. Predicted GATA binding in a MEIS PBA up-binding region is visualised as in the example (a feature matching GATA TF recognition motif on chr5:104257972-104258015 is shown). GATA6 ChIP-seq in PBA verifies this prediction. HOMER was used to cluster and annotate tissue-specific sequence features; features enriched in the different classes of MEIS peaks are matched to TF families with known tissue-specificity (see also Fig 6H). H. Heatmap of the expression of selected TF families, corresponding to cognate recognition motifs identified in MEIS PBA-up, in E11.5 mouse BA2 and PBA. Members of the GATA and TBX families, and the majority of expressed Forkhead TFs are enriched in PBA relative to BA2. Only TFs with expression values > 10 cpm in at least one tissue are shown. I. HOMER de novo motif discovery in HOXA3-specific and HOXA2-specific peaks. HOXA3-specific are HOXA3 peaks excluding peaks overlapping with HOXA2 BA2; similarly, HOXA2-specific are HOXA2 peaks excluding peaks overlapping with HOXA3 PBA. HOMER identifies enrichment of the same motifs enriched in BA-specific MEIS differential binding, Forkhead motif in HOXA3-specific (shaded in green) and BHLH motif in HOXA2-specific subsets (shaded in red). Variations of HD recognition motifs potentially recognized by HOX and attributed by HOMER to PBA-specific TFs NKX and ISL1 in PBA and LHX/DLX in BA2 are also enriched. J. Effects of Forkhead and GATA motifs on HOX and MEIS binding, assessed by *in silico* knockout. CNN MEIS PBA ‘up-binding’ features (Fig 6G) were annotated as HOX, GATA, and Forkhead (see methods). Co-occurring HOX- Forkhead motifs (distance between 1 nt to 100 nt) were selected for *in silico* mutagenesis. Forkhead mutagenesis results in a significant drop in HOXA3 binding in PBA, but shows no average significant effect on HOXA2 in BA2. Similarly, Forkhead mutagenesis significantly decreases Meis PBA binding across most tested sites. In comparison, much weaker effects are predicted on BA1 and BA2 MEIS differential binding. As a negative control, the same procedure was applied to co-occurring HOX-GATA motifs. GATA motif mutagenesis does not show significant average effects on HOX, or MEIS in HOX-bound regions. K. Luciferase activity driven by *Sfrp2* enhancer co-transfected with *Meis* alone (grey bar) and with *Hoxa2* (red empty bars), *Hoxa3* (green empty bars); *Meis* and *Pb*x with *Hoxa2* (red bars) and *Hoxa3* (green bars) in the absence (left half of the graph) and in the presence of *Foxc1* (right half of the graph, as indicated) in NIH3T3 cells. The last two samples contain *Sfrp2* enhancer co-transfected with *Hoxa2* (red empty bars), *Hoxa3* (green empty bars). Adding *Foxc1* to *Hoxa2* or *Hoxa3* with *Meis2* and *Pbx1a* results in the highest activation.

MEIS TFs bind broadly and to largely overlapping locations across different BAs (Fig 6A). In contrast to MEIS ubiquitous occupancy, MEIS binding levels are BA-specific (Fig 6B and 6C) and are associated with distinct biological processes (S6A Fig). We systematically extracted MEIS differential binding across the BAs (S7 Fig) and found that higher MEIS binding levels in a tissue are highly predictive of increased enhancer activity in the same tissue (Fig 6D). Moreover, consistent with MEIS positive effects on transcription [34], genes preferentially expressed in one tissue display higher MEIS binding levels in their vicinity, in a pattern mirroring changes in H3K27Ac across the BAs (Fig 6E). As high MEIS binding levels are tissue-specific and linked to transcriptional activation, we next asked what causes differences in MEIS binding levels across the BAs. Consistent with the finding that HOXA2 enhances MEIS occupancy at selected sites across the genome to activate gene expression [18], regions occupied by HOXA2 in BA2, and by HOXA3 in PBA, display higher enhancer activity if associated with higher, differential MEIS binding levels in the corresponding tissue (Fig 6F). Likewise high MEIS peaks in HOX-positive areas (BA2 and PBA) are highly enriched in sequence features matching HOX-PBX motif [35]. However, HOX co-occupancy only partly accounts for high levels of MEIS binding and acetylation in BA2 and PBAs (S6B Fig), suggesting additional TFs may be involved. TF occupancy *in vivo* occurs in the context of chromatin, which acts as a barrier to TF binding. Multiple TFs that recognize closely spaced binding sites can more effectively compete with nucleosomes and indirectly facilitate each other’s binding. We reasoned that this indirect form of cooperativity between TFs could enhance MEIS binding, resulting in higher MEIS peaks. Therefore, to identify BA-specific TFs that facilitate MEIS binding, we searched for enriched sequence features in MEIS differential binding. CNN models identify enrichment of distinct signature motifs in MEIS differential peaks (Fig 6G), which reflect the distribution of TFs across the BAs (Fig 6H). In support of tissue-specific TFs contributing to MEIS binding levels, GATA6 occupancy in the PBA [30] overlays MEIS peaks, higher in the same tissue *(*S6C Fig*)*. These observations suggest that binding of tissue-specific TFs, in addition to HOX, can locally increase MEIS binding levels.

We noticed that a set of distinctive sequence features of MEIS differential binding in PBA [NKX (HD) and FOX (Forkhead) motifs] and BA2 [(basic helix-loop-helix (bHLH) recognition sites] (Fig 6I) are also enriched in HOXA3- and HOXA2-specific peaks, respectively, suggesting that HOX and tissue-specific TFs may collaborate in binding with TALE. We decided to focus on FOX TFs, as most *Fox* genes are expressed at higher levels in PBA than BA2 (Fig 6H). Consistent with the three factors cooperating on chromatin, HOX and FOX recognition sites co-occur in the same differential MEIS peaks (S6D and S6E Fig). The higher levels of FOX TFs in the PBA, relative to BA2, predict FOX TFs to have stronger effects on HOXA3 and MEIS binding in PBA. CNN can be trained to predict binding intensities merely based on DNA nucleotide sequence, and the learned model can be used to predict changes in binding intensities upon random dinucleotides mutations in TF binding motifs (in silico mutagenesis). Indeed, in silico mutagenesis predicts mutations in FOX TF recognition sites to affect binding of both HOXA3 and MEIS in PBA, but not HOXA2 and MEIS in BA2 (Fig 6J, S6F Fig). In contrast, mutagenesis of GATA motifs (enriched in MEIS differential peaks, but not in HOX peaks) does not appear to affect HOX-MEIS binding (Fig 6J). Synergistic effects of FOX and HOX have been identified in the mouse central nervous system [36]. In luciferase assays, addition of FOXC1, HOX and MEIS/PBX increases transcriptional activation driven by the *Sfrp2* distal region (co-occupied by HOX and FOXC1 in the BAs) [18] (Fig 6K). While naked DNA in transfection assays does not recapitulate TF cooperativity on chromatin, these results imply that TALE FOX and HOX TFs bind their predicted binding sites and together, contribute to transcriptional activation. Moreover, the presence of FOXC1 is sufficient to enhance MEIS/HOX transcriptional activation of *Sfrp2* enhancer, suggesting that cooperation between these TFs could partly compensate for lack of PBX (Fig 6K).

These results identify (direct or indirect) cooperativity with tissue-specific TFs as an additional mechanism for HOX selectivity. By enhancing MEIS binding (residence time on chromatin), tissue-specific TFs also contribute to promote the stability of HOX-MEIS complexes in specific tissues. The distribution of tissue-specific motifs in HOX peaks suggests this mechanism accounts for HOX selectivity at a subset of enhancers, rather than being a general mechanism of HOX enhancer selection (S6G Fig). We propose that HOX and tissue-specific TFs (alone and in combination) increase TALE TF binding affinity and residence time at selected locations, identified using their sequence recognition motifs. As high levels of MEIS are associated with increased enhancer activity and gene expression, longer MEIS residence time at selected locations may translate into BA-specific transcriptional outputs.

## Discussion

HOX TFs contain a HD, which display highly similar sequence recognition properties and is shared by hundreds of TFs, yet they instruct diverse, segment-specific transcriptional programs along the antero-posterior axis of all bilaterian animals. By profiling HOXA2 and HOXA3 binding in their physiological domains, we identify three main determinants of HOX-selective binding across the genome: 1) recognition of unique variants of the HOX-PBX motif; 2) differential affinity at ‘shared’ HOX-PBX motifs and; 3) presence of additional tissue-specific, non-TALE, TFs. These mechanisms (with the possible exception of the first) are expected to generate quantitative (rather than qualitative, i.e. binding/no binding) differences in the relative levels of HOX/TALE occupancy on commonly bound regions. Such quantitative changes are a feature of continuous networks [37], in which TFs bind a continuum of functional and non-functional sites and regulatory specificities derive from quantitative differences in DNA occupancy patterns.

HOX paralog-selective binding occurs in cooperation with TALE. The high degree of HOX and TALE interaction flexibility, mediated by paralog-specific protein signatures, has been proposed to generate paralog-specific functions of HOX TFs [38]. Here, by defining the *in vivo* repertoire of HOX occupied sites, we identify DNA sequence as an additional determinant of HOX-TALE functional specificity *in vivo*. This finding is consistent with the mechanism of latent specificity described for *Drosophila* Hox/Exd (PBX) interaction [39] and *in vitro* observations that HOX TFs bind longer, more specific sequence motifs in the presence of TALE. However, the effects of TALE on HOX binding *in vivo* go beyond the refinement of HOX binding sites as, at least in the BA context, binding with TALE appears to be a requirement for loading HOX on chromatin. Our observations indicate that HOXA2-A3 overwhelmingly recognize genomic sites that are enriched in HOX-PBX motifs and are also occupied by TALE TFs *in vivo*. Therefore, TALE provides a platform for HOX to bind; selectivity enables HOX paralogs to preferentially bind different subsets of this common platform. In agreement with our finding that BA-specific chromatin states do not seem to play a role in HOX target site selection, TALE platform is largely similar across BA1-2-PBA.

**Fig 7:**
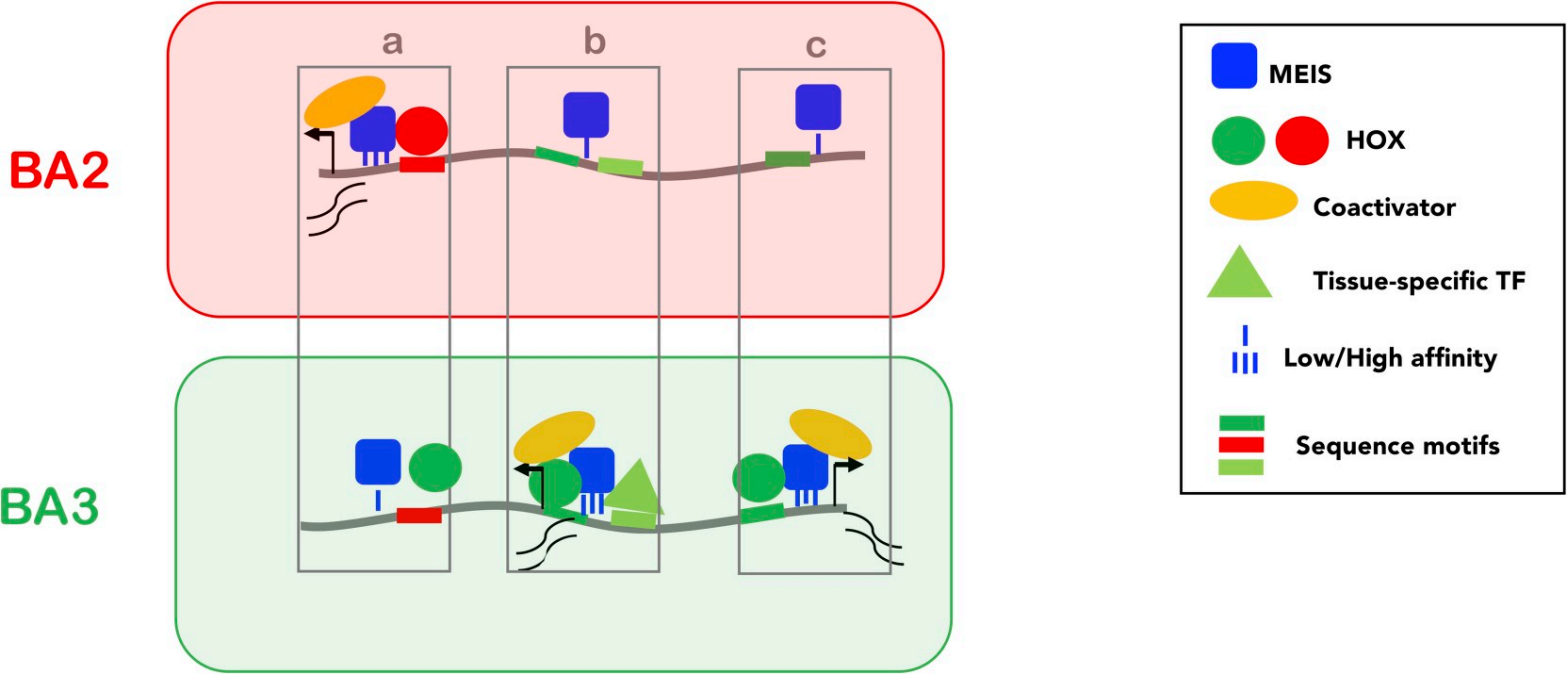
Model. Low affinity, widespread binding of MEIS (blue square) defines a large subset of accessible chromatin (grey line) for activation (PBX is not shown as PBX and MEIS binding almost entirely overlap). Direct cooperativity with HOX (A2 and A3, red and green circles respectively) and/or indirect cooperativity with tissue-specific TFs (triangle) increase MEIS binding affinity and residence time; prolonged residence time of MEIS at enhancers promotes recruitment of general co-activators (yellow) and activation of transcription. HOX paralogs preferentially bind different subsets of MEIS occupied regions, resulting in differential transcription. Three examples of BA-specific transcription are shown. In **a**, the red site is bound with higher affinity by HOXA2 than HOXA3, resulting in the formation of a more stable HOX-TALE complex on DNA and a (higher) transcriptional output in BA2. Conversely, in **c**, the green site is only recognized by HOXA3, leading to high affinity MEIS binding only in PBA, and to PBA-specific transcription. In **b**, the effect of HOXA3 is potentiated by a PBA-specific TF binding in the vicinity. Co-binding with tissue-specific TFs may positively contribute to HOX-MEIS cooperativity by competing with nucleosome for DNA binding, especially at HOX and/or MEIS low affinity sites. These mechanisms result in BA-specific transcription.

What is the functional significance of HOX-TALE interaction on chromatin and how does it contribute to paralog-specific transcriptional programs? Many examples from animal development indicate that transcriptional regulation is mediated by distinct combinations of TFs. TALE TFs operate as a hub, which assists combinatorial assembly of TF complexes. TALE platform enables HOX function to be modulated by other TFs; in doing so, it integrates positional signals (encoded by HOX) and local inputs (provided by cell type-/tissue-specific TFs) into defined transcriptional outputs. While it is possible that MEIS and PBX facilitate access of diverse TFs to relatively inaccessible chromatin, MEIS TFs differ from conventional pioneer TFs, which function to open chromatin regions but are not directly involved in enhancer activation [40,41]. Remarkably, independently of the type of TF involved (HOX or other tissue-specific TFs), positive changes in MEIS binding result in a functional effect, i.e. increased enhancer activity. High instances of MEIS binding are typically tissue-specific and highly correlated with enhancer activity. In fact, differential MEIS binding in a specific BAs is generally a very good predictor for matching changes in enhancer activity in the same tissue. Based on our observations and the well-established role of MEIS in transcriptional activation [34,42,43], we propose a model of transcriptional activation, where TALE (MEIS) TFs function as broad or general activators and HOX paralog selectivity is mainly directed at harnessing TALE functional activity at selected locations. Using their recognition motifs, HOX and/or tissue-specific TFs select specific MEIS binding locations, where they stabilize MEIS binding to generate precise functional outputs, or patterns of enhancer activation (Fig 7). Interestingly, MEIS2 interacts with PARP1 [43], a large enzyme capable of triggering phase condensation [44]. Increasing MEIS residence time (as a result of the cooperation with HOX and other TFs) may favour PARP1 recruitment at selected loci and, in turn, generate the liquid-liquid phase transitions observed to promote gene activation [8,45].

Because high instances of MEIS binding are typically associated with combinatorial TF binding, a precise identification of the critical steps for enhancer activation, and their sequential order, remains problematic. For similar reasons, MEIS and PBX shared genomic occupancy complicates dissecting their respective contributions to enhancer binding and activation. In addition to TALE, numerous other TFs are broadly, if not ubiquitously expressed during development, yet their inactivation results in tissue-specific phenotypes. It is tempting to speculate that similar principles of TF functional connectivity could explain other transcriptional networks, i.e. that cell type- tissue-specific regulators harness the activation abilities of broadly expressed TFs to generate cell type -specific gene expression programs.

## Material and methods

### Ethics statement

Experiments using animals were performed in accordance with legislation in the UK Animals (Scientific Procedures) Act of 1986 (PPL 70/8858 to Graham Morrissey). All procedures on zebrafish adults and embryos were approved by the University of Massachusetts Institutional Animal Care and Use Committee (IACUC).

### Animal experiments

CD1 mice were time-mated to obtain BA2 or PBA from E115 embryos. Mouse experiments were carried out under ASPA 1986. Wild type zebrafish were raised in the University of Massachusetts Medical Center Zebrafish Facility. Embryos and adult zebrafish were maintained under standard laboratory conditions. Enhancers were amplified from mouse genomic DNA using the primers (listed in S3 Table), cloned into pCR8/GW/TOPO vector (Life Technologies) and recombined using the Gateway system (Life Technologies) to an enhancer test vector that includes a strong midbrain enhancer (Minitol2-GwB-zgata2-GFP-48, a kind gift from JL Skarmeta) as an internal control. Fertilized zebrafish embryos were collected from natural spawnings. Plasmid DNA was injected into the cytoplasm of one-cell stage embryos. Injected embryos were visualized intermittently by fluorescence microscopy up to 48 hr post fertilization to identify transgenic carriers. These were raised to adulthood, outcrossed to wildtype fish and the resulting F1 embryos were scored for GFP expression in order to generate stable transgenic lines.

### Next-generation sequencing data and downstream analyses

ChIP-seq was performed as described [30] using rabbit polyclonal antibodies targeting HOXA3 (non-conserved N-terminal amino acids 24 to 180), HOXA2 [46], PBX1-2-3-4 (sc-25411X, Santa Cruz), MEIS1/2 (sc-10599X, Santa Cruz) and rabbit IgG (Millipore). DNA was recovered from two independent ChIP-seq experiments and purified using DiaPure columns (Diagenode). Enrichment was validated by SYBR green quantitative PCR (qPCR) using primers listed in S3 Table. DNA libraries were constructed using the MicroPlex Library Preparation Kit v2 (Diagenode) and sequenced with the Illumina next generation sequencing platform. ChIP-seq experiments were analysed using Trimmomatic for trimming [47], Bowtie2 for aligning to the mouse genome (mm9) [48], samtools [49] to remove the aligned reads with a mapping quality Q30 and MACS2 for peak calling [50] with default narrow peak calling setting for TFs and broad peak calling setting for histone modification marks. ‘*findMotifGenome’* module of the HOMER package was used to detect *de novo* motif in 200nt summit regions [24]. Venn diagrams were generated using 200nt peak summits with an overlap of at least 1nt. GREAT standard association rule settings [25] was used to associate ChIP-seq peaks with genes and uncover events controlled by TF binding. DiffBind [51] was used to re-center MEIS and H3K27ac peaks across BA1, BA2 and PBA (S7 Fig) and calculate RPKM values and raw counts in the re-centered regions. edgeR generalized linear model (GLM) method with TMM normalization [52] was used to select differential peaks and calculate fold change in MEIS binding and H3K27ac across BAs used to generate boxplots and scatterplots. Gene expression CPM values and differential gene expression at E10.5 and E11.5 were derived from [18,30]. ggplot2 package [53] was used to generate CPM values heatmap. GALAXY [54], Bioconductor GenomicRanges package [55], and Bioconductor ChIPpeakAnno package (https://www.bioconductor.org/packages//2.10/bioc/html/ChIPpeakAnno.html) were used to intersect, modify and visualize genomic coordinates. Bioconductor Biostring [56] was used to locate fixed motif sequences in the binding regions. Distance between HOX and MEIS binding regions was calculated using GenomicRanges package and plotted with ggplot2. The Kernel density distribution of MEIS fold enrichment in HOX binding regions vs non-HOX binding regions were calculated by R kernel density distribution estimation (R core team 2013) and plotted with ggplot2.

All RNA-seq and ChIP-seq datasets are available on ArrayExpress.

### Convolutional neural network models and *in silico* mutagenesis

MEIS differential sequence features are detected by recently published differential convolutional neural network (CNN) structure [35]. For *in silico* binding site knockout we trained a CNN model for multitask regression of MEIS and HOX RPKM binding level. The CNN was trained by transfer learning, using convolution parameters from a previously published 1-convolutional layer MEIS RPKM model [35]. Convolutional filters were transferred to a new model, which was then trained on a subset of MEIS regions also bound by HOX, to simultaneously predict log2RPKM values in 2 replicates of HOXA2 in BA2, 2 from HOXA3 in PBA, and one replicate of MEIS in BA1, BA2 and PBA. The training data consisted of 6795 regions of 600nt with HOX binding predicted by MACS2 in any tissue. The regression model was subsequently used to predict the change in RPKM values after binding site erasure. For simulated genomic knockout, a 25nt site containing each feature was replaced by random di-nucleotides from the remaining part of the region and RPKM levels were predicted. Random replacement was repeated 100 times for each feature, averaging the predicted RPKM change. To select candidate features for erasure, MEIS PBA up-binding features were first obtained from the previously published 3-task parallel model and subsequently filtered. Sites of HOXA3 and GATA were required to contain consensus motif “TGATNNAT” and “WGATAA” respectively, with no mismatch allowed. Forkhead sites were selected based on long distinct k-mers, derived from KSM motif representation method [57], namely exact matches to any of the following sequences: “AAAATAAACA", "AAAAATAAAC", "AATAAATCAA", "ATNAATCAACA", "AAATAAACAC", "ATAAATCAAC","GAAAATAAAC", "CAAAATAAAC", "AAAATAAACT", "AAATAAACAA". These candidate sites were identified within a +/- 250nt window centred on HOXA3 and GATA6 ChIP-seq peak summits, FE of replicates was combined with edgeR [52] and a Poisson test was performed as in MACS2 using false discovery rate (FDR) cutoff = 0.05. Only Forkhead and GATA motifs that did not contain internal matches to HOX-PBX motif were selected. Subsets of GATA and Forkhead sites located within +/- 100nt from a HOX-PBX sites were selected for mutagenesis.

### Single cell analysis

Single cell transcriptomic data from [28] was retrieved from GEO under accession code GSE129114. Low-abundance genes were filtered according to [58]. Read counts are normalized according to [59]. Highly variable genes (HVGs) were selected using ‘modelGeneVar’ from scran bioconductor package [60], and used for clustering and dimension reduction. Clustering was done using Hierarchical clustering function hclust in R with ward.D2 method [61]. Dimension reduction was performed using with the ‘Rtsne’ function in R.

### Electrophoretic mobility shift assays

Probes were made from primers with 5’ ATO700, and purified with QIAGEN PCR purification kit (Qiagen). Proteins were generated using TnT Quick Coupled Transcription/Translation System (Promega) and the following plasmids: pcDNA3-Hoxa2, pcDNA3-Hoxa3, pcDNA3-Meis2, containing mouse coding sequences for *Hoxa2*, *Hoxa3* and *Meis2* (isoform 1), cloned into pcDNA3 (Invitrogen); pcDNA3-PBX1a is a gift from Francesco Blasi. Reactions (4% Ficoll, 20mM HEPES, 37.5mM KCl, 1mM DTT, 0.1mM EDTA, 2μg Poly dI.dC, 16ng probe, and 2μl of TNT extracts in total volume of 10μl) were mixed by gentle flicking, and incubated at room temperature for 12 minutes before being run on 4% acrylamide gel at 70V in 0.5X TBE. Bands were quantified using ImageJ (version 1.52a). Intensity plots for each lane were generated by selecting the same region across all the lanes. Individual band intensities were plotted as histograms areas and quantified by the software. Values in triplicate were averaged and, unless stated otherwise, adjusted to the band intensity of the probe bound by PBX/MEIS in the absence of any HOX. Corresponding numerical data are provided as supporting information (data summary).

### Luciferase assay

*Meis2* and *Zfp703* enhancers were amplified from mouse genomic DNA using primers listed in S3 Table cloned into pCR8/GW/TOPO vector (Life Technologies) and recombined using the Gateway system (Life Technologies) into pGL4.23-GW (a gift from Jorge Ferrer; Addgene plasmid # 60323; http://n2t.net/addgene:60323RRID:Addgene_60323). Enhancers were co-transfected with pcDNA3, pcDNA3-Hoxa2, pcDNA3-Hoxa3, pcDNA3-Meis2, pcDNA3-PBX1a (described above) and pcDNA3-Hoxd3 generated by GenScript. NIH3T3 cells were grown in DMEM (D6429) supplemented with 10% FBS and 5% penicillin/streptomycin, and seeded in 24-well plates at 100,000 cells in 1ml. Cells were transfected with GeneJuice Transfection Reagent (Novagen), using 250ng luciferase plasmid and 300ng pcDNA3 plasmids per well. Cells were harvested 24 hours after transfection and luciferase measured using Luciferase Assay System and the GloMax Multi-Detection System (Promega). Corresponding numerical data are provided as supporting information (data summary).

### Antibody validation

Gateway entry vectors for mouse *Hoxb1* and *Hoxb2* [62], human *HOXA3* and *HOXC4* (http://horfdb.dfci.harvard.edu/hv7/) were used to generate mammalian expression vectors for FLAG-HOX (v1899 destination vector) using the gateway technology [63]. Gateway expression vectors for pExpFLAG-Hoxa1 and pExpFLAG-Hoxa2 are described in [64,65]. HEK293 cells were grown at 37°C using DMEM (D6429) supplemented with 10% FBS, 5% penicillin/streptomycin, and 5% L-glutamine. Cells were seeded in 6-well plates at 400,000 cells/well and transfected 24 hours after plating using 1μg of HOX plasmid constructs and Fugene6 (Promega) according to the manufacturer's instructions. Proteins were collected 48 hours after transfection, boiled in Laemmli buffer, run on SDS-page and visualized using anti-FLAG (M2) (#F1804, Sigma), HRP-conjugated anti-β-ACTIN (#A3854, Sigma) and anti-Hoxa3 antibody (1:2000) and HRP-conjugated secondary antibodies.

### Co-precipitation experiments

Coding sequences for MEIS1b and MEIS2.1 were cloned in pEnt plasmids, confirmed by DNA sequencing and used to generate pExp mammalian expression vectors for GST-tagged proteins with the pDest-GST N-terminal destination vector using the gateway technology [66]. HEK293 cells were transfected as above, using 500ng each of FLAG/GST constructs per well. Proteins were collected 48 hours after transfection and co-precipitation performed as described in [62].

## Supporting information

S1 Fig A. Specificity of HOXA3 polyclonal antibody. Western blot using FLAG and HOXA3 antibodies, as indicated. HOXA3 antibody recognizes HOXA3 and does not cross react with mouse HOXA1, HOXA2 and human HOXB1, HOXB2 and HOXC4. Plasmids containing *HOX*-FLAG cDNAs were transfected in HEK293 cells. B. Comparison of HOXA3 ChIP-seq replicates in E11.5 PBA (FE≥10). The replicate with larger genome coverage contains most (80%) of the second replicate’s peaks and is used in all subsequent analyses. C. Top three most significant motifs identified in HOXA3 peaks using *de novo* motif discovery. D. Comparison of HOXA2 ChIP-seq replicates in E11.5 BA2. The smallest replicate was sequenced using the Illumina platform and is used for subsequent analysis; it largely (73%) overlaps HOXA2 ChIP-seq run on the Solid platform (Donaldson et al., 2012). E. Sequence logo of the top three most significant motifs identified in HOXA2 peaks. F. Frequency of HOX-PBX motifs in HOXA3 and HOXA2 peaks. G. Percentage of HOXA2 (red) and HOXA3 (green) peaks containing TGATNNAT motifs, with no mismatch (triangles) or 1nt mismatch (circles) allowed. Increasing numbers of top HOX peaks, ordered by decreasing FE, are plotted on the x axis. For both HOXA2 and HOXA3, the percentage of peaks containing a perfect match (no mismatch) to TGATNNAT decreases with relaxing FE. The opposite trend is observed for the distribution of TGATNNAT motifs with 1nt mismatch allowed. H, Enrichment of HOXA2 regulated genes (red fraction) in the top biological processes and mouse phenotypes identified by GREAT; the remaining fraction corresponds to genes associated with HOXA2 peaks, but not significantly dysregulated in HOXA2 mutant embryos. The number of genes in each category and the corresponding p value (Fisher’s exact test) are indicated.
(PDF)

S2 Fig A. Percentage of TGATNNAT variants in top 250 or in top 250 to 2000 HOXA2 and HOXA3 peaks, as indicated. HOX-selective binding is more evident in the fraction of high-confidence peaks, suggesting that analysis of whole ChIP-seq experiments may mask effective HOX specificity. B. Distance between TGATNNAT (HOX-PBX) and TGACA (MEIS) in top 250 HOXA2 (red) and HOXA3 (green) peaks. Most TGACA occur at <20 nt from a HOX-PBX site. C. Quantification of MEIS/PBX/HOX (trimeric) complexes binding to the wild-type and mutant *Sulf2* probe. Complexes are colour-coded according to the presence of HOXA2 (red boxes) and HOXA3 (green boxes). Values (raw data) were obtained from three independent experiments. Binding of HOXA3 to the wild-type *Sulf2* probe is significantly higher relative to HOXA2, while no significant difference is detected between HOXA3 and HOXA2 binding to the *Sulf2* mutant probe. One-way ANOVA with post-hoc Tukey HSD (Honestly Significant Difference) test: * = p<0.05.
(PDF)

S3 Fig A. UCSC tracks of HOXA2, HOXA3, PBX, MEIS binding and H3K27 acetylation profiles in BA2 (red) and PBA (green) at the *Zfp703* locus. Strong HOX and TALE binding is observed in both tissues, with higher acetylation levels in BA2. B. Luciferase activity driven by *Zfp703* enhancer co-transfected with *Hoxa2* (red bar), *Hoxa3* (green bar), *Hoxa2-a3HD* (red empty bar), *Hoxa3-a2HD* (green empty bar), *Meis2* and *Pbx1a* (grey bar) expression vectors, alone and in combination, in NIH3T3 cells. Addition of MEIS/PBX amplifies the difference in HOXA2 and HOXA3 activation abilities. Changing the HOX-PBX site (shown in C) reduces HOX-TALE activation to the levels observed with TALE alone (empty bars). Values represent fold activation over basal enhancer activity and are presented as the average of at least two independent experiments, each performed in triplicate. Error bars represent the SEM. One-way ANOVA with post-hoc Tukey HSD test: * = p<0.05; *** = p<0.0005. C. Sequence of *Zfp703* wild-type and mutant enhancer encompassing HOX-PBX and MEIS sites. HOX-PBX and MEIS motifs are underlined. Nucleotide substitution in the HOX-PBX site are shown in red. D. Luciferase activity driven by *Meis2* enhancer co-transfected with *Hoxa2* (red bar), *Hoxa3* (green bar), *Hoxd3* (green bar), *Meis2* and *Pbx1a*, alone and in combination. When co-expressed with MEIS and PBX, HOXA2 shows higher activation capacity than HOX paralog 3. Statistical analysis was performed as in B. EF. HOXA2 (E, red arrow) and HOXA3 (F, green arrow) weakly bind the *Meis2* probe. MEIS and PBX bind DNA together (black arrow). HOXA2 (E) or HOXA3 (F) do not bind the *Meis2* probe with either MEIS or PBX alone. Addition of HOXA2 and HOXA3 to MEIS/PBX results in a trimeric protein complex (arrowheads in E and F). G. Quantification of MEIS/PBX and HOX binding to *Meis2* probe. Binding of MEIS/PBX (dimeric) and MEIS/PBX/HOX (trimeric) complexes is expressed as relative percentage to PBX/MEIS binding to *Meis2* probe in the absence of any HOX. Dimeric and trimeric complexes are colour-coded according to the presence of HOXA2 (red boxes), HOXA2-A3HD (dark red boxes), HOXA3 (green boxes) and HOXA3-A2HD (dark green boxes). Values were obtained from at least three independent experiments. HOXA2 binds the *Meis2* probe with higher affinity with PBX/MEIS, relative to HOXA3. Accordingly, HOXA2 shifts more of the PBX/MEIS dimer into a trimeric complex, relative to HOXA3. A similar behaviour is observed with chimeric HOX (*** = p<0.0005; * = p<0.05, one-way ANOVA with post-hoc Tukey HSD test). HI. Quantification of HOX/MEIS/PBX trimeric (H) and MEIS/PBX dimeric (I) binding to *Meis2* probe shown in Fig 3H. Binding of MEIS/PBX (dimeric) and MEIS/PBX/HOX (trimeric) complexes is expressed as relative percentage to PBX/MEIS binding to *Meis2* probe in the absence of any HOX. The total HOX dose is unvaried and corresponds to 100%; HOXA2 and HOXA3 are translated alone and co-translated with each other at different ratios, as follows: 100% HOXA2; 75% HOXA2 + 25% HOXA3; 50%HOXA2 + 50% HOXA3; 25% HOXA2 + 75% HOXA3; 100% HOXA3. Colour coded % are indicated below the x-axis; HOXA2 is red and HOXA3 is green. HOXA2 forms a significantly stronger trimeric complex than HOXA3 at all comparable concentrations, except 25% (difference not significant). Values were obtained from three independent experiments. I. PBX/MEIS complex is significantly reduced in the presence of all HOX. HOXA2 causes a significantly greater reduction than HOXA3. One-way ANOVA with post-hoc Tukey HSD test: * = p<0.05; **p<0.005; *** = p<0.0005. J. Same experiment as in Fig 3G, run on a lower percentage gel to resolve slower retarded bands. No apparent HOXA2/HOXA3/PBX/MEIS complexes can be detected as slower bands above HOXA3 trimeric complex. K. Mutations in MEIS and HOX/PBX recognition sites affect MEIS/PBX and HOX/MEIS/PBX binding to *Meis2* probe. Arrow and arrowheads indicate dimeric and trimerix complexes, respectively; asterisks mark lanes containing a detectable trimeric complex. Representative gels are shown; each independent gel contains PBX/MEIS binding to *Meis2* wild-type probe as a reference. Mutant probes are indicated on the top and named as in Fig 3H; quantifications of MEIS/PBX dimers are shown in Fig 3I.
(PDF)

S4 Fig A. tSNE (T-distributed Stochastic Neighbour Embedding) of E10.5 post-otic cranial neural crest single cell RNA-seq (Soldatov et al. 2019) clusters cells into four main clusters. B. Two clusters (green and purple ovals in AB) exhibit high-expression of *Hoxa2* and *Hoxa3*. Count of cell expressing *Hoxa2* and *Hoxa3* in the green and purple clusters shows that the majority of cells co-express *Hoxa2* and *Hoxa3*, more than what is expected by chance (Fisher exact test). C. Quantification of HOX/MEIS/PBX binding to *Meis2* probe shown in Fig 4E. Each HOX is co-translated with PBX/MEIS alone or with the other HOX. The highest HOX dose corresponds to 100%; HOXA2 (red) is translated at progressively decreasing levels, either alone, or co-translated with progressively increasing levels of HOXA3 (green) as indicated on the x-axis. At 50% and 25% levels, HOXA2 forms a significantly stronger trimeric complex (red arrow) on its own than when HOXA3 is added.
(PDF)

S5 Fig A. Whole Venn diagram of HOXA3, MEIS and PBX binding overlap, cropped in Fig 5A. Most MEIS and PBX peaks do not overlap with any HOXA3 peaks. B-D. Kernel density plots of MEIS peaks relative to FE. MEIS binding is sorted into peaks not overlapping HOX (lighter colour) and peaks overlapping HOX as indicated (darker colour). HOX and MEIS ChIP-seq in mouse embryonic stem cells (HOXA1), BA2 (HOXA2) and hematopoietic stem cells (HOXA9) were used in this analysis.
(PDF)

S6 Fig A. GREAT analysis of MEIS peaks higher in BA2 (relative to PBA, red) and PBA (relative to BA2, green). Fold change ≥5 across BAs is used as a cutoff. Differential MEIS peaks are associated with genes involved in different biological processes (top 10 categories are shown). BC. Fold change (PBA versus BA2) in MEIS binding and H3K27ac levels at HOX and GATA peaks. B. HOXA3 binding in PBA (green) and HOXA2 binding in BA2 (red) is associated with increased MEIS binding and H3K27ac in the corresponding tissues. C. Similarly, GATA6 binding in PBA is associated with increased MEIS and H3K27ac in PBA. DE. HOMER motif search in the vicinity of HOX/PBX sequence features identified by CNN (+/-100 nt around TGATNNAT, as shown in D) identifies enrichment of Forkhead motifs in MEIS higher PBA. Other enriched flanking motifs include MEIS and HD motifs. F. Example of HOX and Forkhead features, identified by CNN in a HOX- bound region. The RPKM feature tracks show combined RPKM change caused by mutating each nucleotide to its alternatives. Features of two experimental replicates for HOXA2 in BA2 and two replicates of HOXA3 in PBA are predicted by a jointly trained multitask model, trained by transfer learning from a MEIS RPKM model. Below, normalised cross-correlation of region sequence with HOX and Forkhead position weight matrix (PWM) obtained by k-mer counting is shown. Nucleotides in HOX and Forkhead motifs show increased sensitivity to perturbation, compared to their surrounding sequences. G. Motifs occurrence in top 250 HOXA2 and HOXA3 peaks (200 nt summits). Frequencies of single and co-occurring motifs are expressed as percentages and colour-coded in the bar plot. Square brackets indicate bHLH (CATCTG) in HOXA2 and Forkhead (TACAAA) in HOXA3 peaks. Motif frequency is calculated using IUPAC letters, as indicated.
(PDF)

S7 Fig Identification of MEIS differential binding. MACS peaks from MEIS ChIP-seq experiments in BA1, BA2 and PBA with replicates were recentered using DiffBind (see methods), and pairwise fold changes and differential binding regions were computed by edgeR as described (Phuycharoen et. al. 2019). Pairwise differential regions were combined into MEIS BA1 up, BA1 down, BA2 up, BA2 down, PBA up, and PBA down regions. Differential sequence motifs were detected as described (Phuycharoen et. al. 2019). Differential H3K27ac regions and fold changes were computed in the same manner.
(PDF)

S1 Table Regions bound by Hoxa3 in PBA, Fold Enrichment (FE)>10. (CSV)

S2 Table Regions bound by Hoxa2 in BA2, FE>10. (CSV)

S3 Table List of primers used in this study. (DOCX)

S1 Data Numerical data relative to electrophoretic mobility shift assays and luciferase assays. (XLSX)
