# Supplementary figures and images for: HOX paralogs selectively convert binding of ubiquitous transcription factors into tissue-specific patterns of enhancer activation
Source: PLoS Genet. 2020 Dec 14;16(12):e1009162. doi: 10.1371/journal.pgen.1009162 (PMC7769617; doi:10.1371/journal.pgen.1009162)

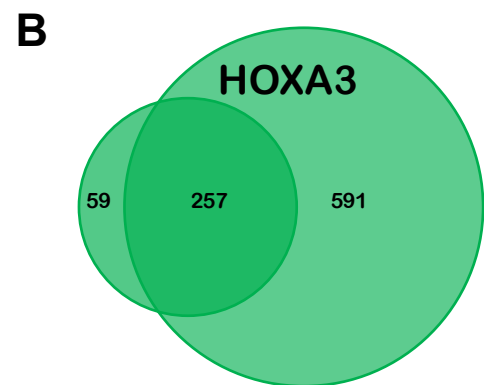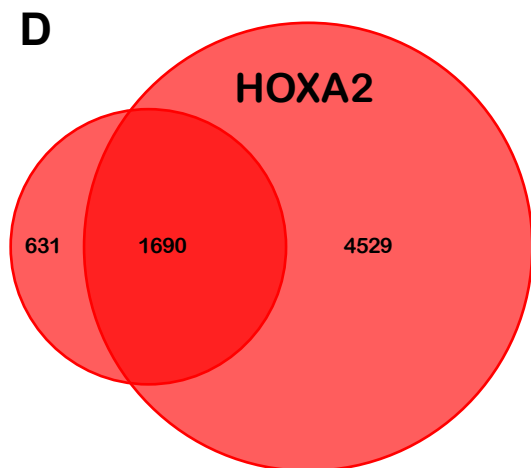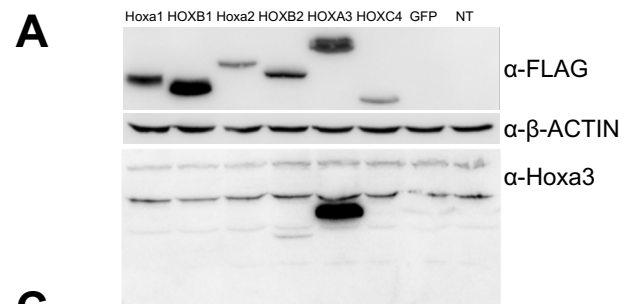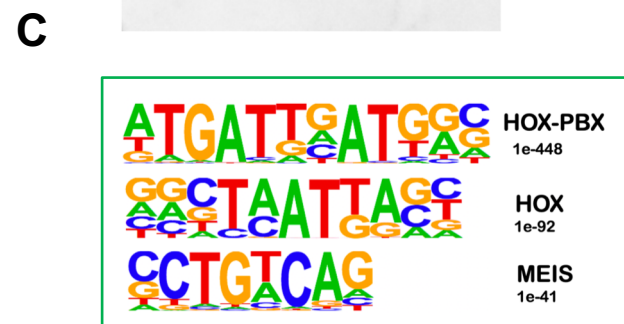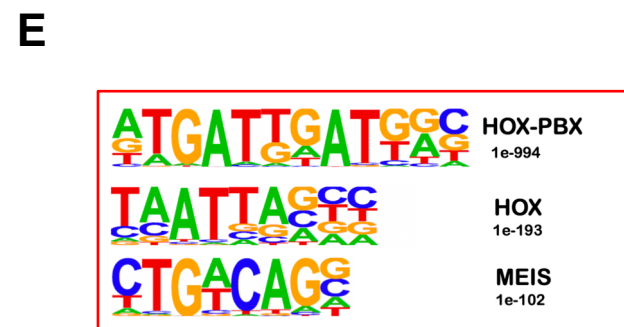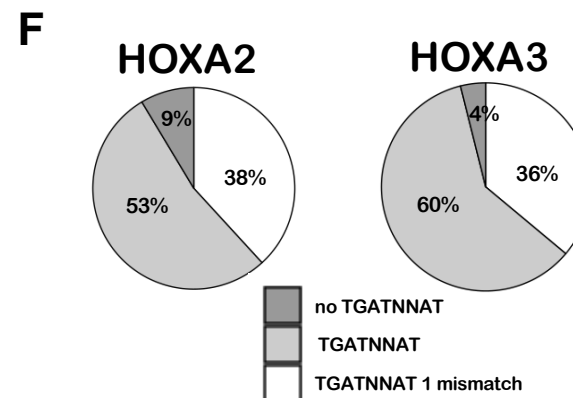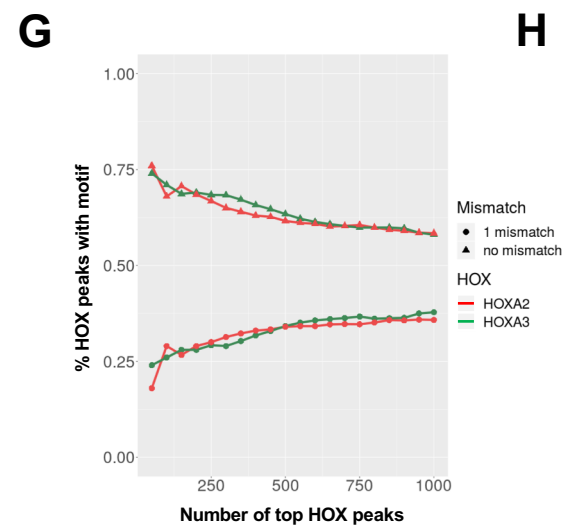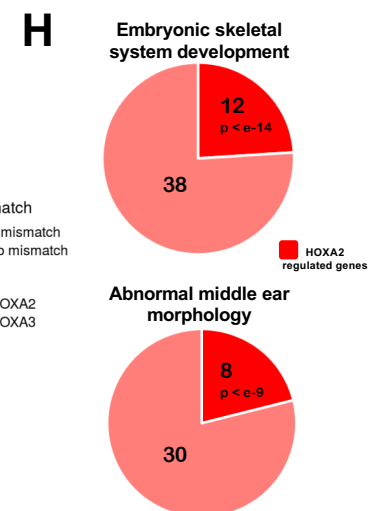

Supplement: S1 Fig — A. Specificity of HOXA3 polyclonal antibody. Western blot using FLAG and HOXA3 antibodies, as indicated. HOXA3 antibody recognizes HOXA3 and does not cross react with mouse HOXA1, HOXA2 and human HOXB1, HOXB2 and HOXC4. Plasmids containing HOX-FLAG cDNAs were transfected in HEK293 cells. B. Comparison of HOXA3 ChIP-seq replicates in E11.5 PBA (FE≥10). The replicate with larger genome coverage contains most (80%) of the second replicate’s peaks and is used in all subsequent analyses. C. Top three most significant motifs identified in HOXA3 peaks using de novo motif discovery. D. Comparison of HOXA2 ChIP-seq replicates in E11.5 BA2. The smallest replicate was sequenced using the Illumina platform and is used for subsequent analysis; it largely (73%) overlaps HOXA2 ChIP-seq run on the Solid platform (Donaldson et al., 2012). E. Sequence logo of the top three most significant motifs identified in HOXA2 peaks. F. Frequency of HOX-PBX motifs in HOXA3 and HOXA2 peaks. G. Percentage of HOXA2 (red) and HOXA3 (green) peaks containing TGATNNAT motifs, with no mismatch (triangles) or 1nt mismatch (circles) allowed. Increasing numbers of top HOX peaks, ordered by decreasing FE, are plotted on the x axis. For both HOXA2 and HOXA3, the percentage of peaks containing a perfect match (no mismatch) to TGATNNAT decreases with relaxing FE. The opposite trend is observed for the distribution of TGATNNAT motifs with 1nt mismatch allowed. H, Enrichment of HOXA2 regulated genes (red fraction) in the top biological processes and mouse phenotypes identified by GREAT; the remaining fraction corresponds to genes associated with HOXA2 peaks, but not significantly dysregulated in HOXA2 mutant embryos. The number of genes in each category and the corresponding p value (Fisher’s exact test) are indicated. (PDF) [file pgen.1009162.s001.pdf]

**A**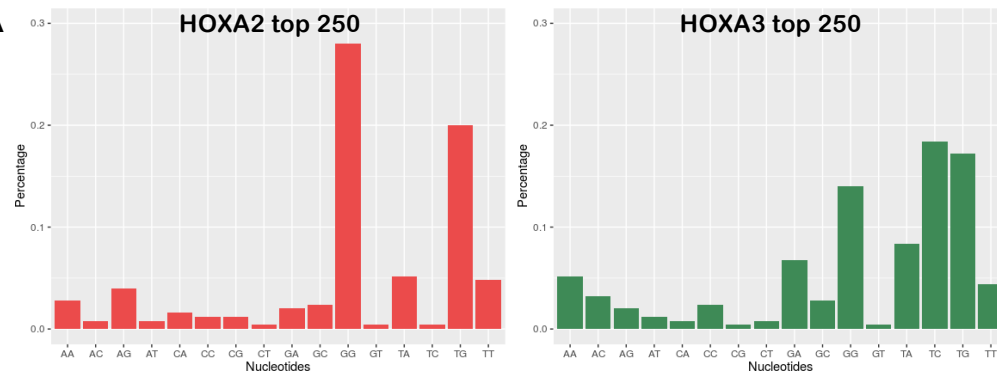**B**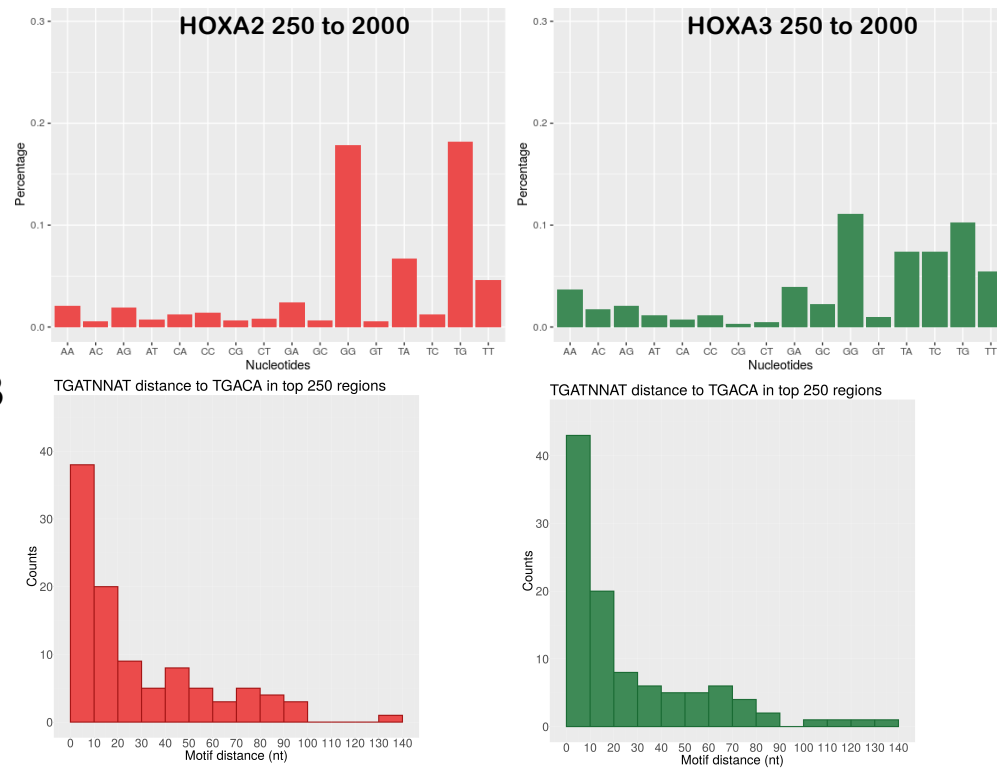**C**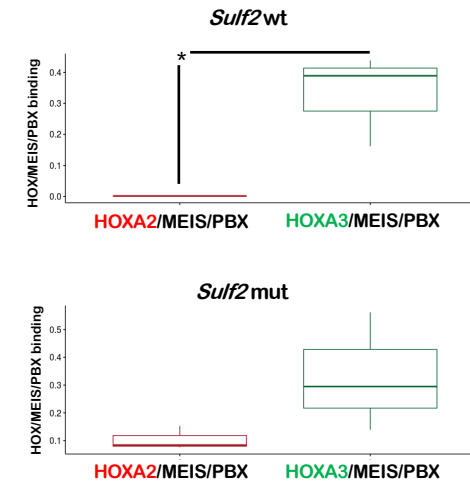**D**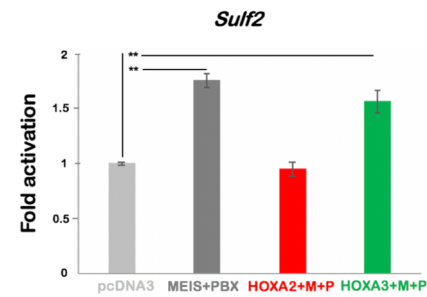

Supplement: S2 Fig — A. Percentage of TGATNNAT variants in top 250 or in top 250 to 2000 HOXA2 and HOXA3 peaks, as indicated. HOX-selective binding is more evident in the fraction of high-confidence peaks, suggesting that analysis of whole ChIP-seq experiments may mask effective HOX specificity. B. Distance between TGATNNAT (HOX-PBX) and TGACA (MEIS) in top 250 HOXA2 (red) and HOXA3 (green) peaks. Most TGACA occur at <20 nt from a HOX-PBX site. C. Quantification of MEIS/PBX/HOX (trimeric) complexes binding to the wild-type and mutant Sulf2 probe. Complexes are colour-coded according to the presence of HOXA2 (red boxes) and HOXA3 (green boxes). Values (raw data) were obtained from three independent experiments. Binding of HOXA3 to the wild-type Sulf2 probe is significantly higher relative to HOXA2, while no significant difference is detected between HOXA3 and HOXA2 binding to the Sulf2 mutant probe. One-way ANOVA with post-hoc Tukey HSD (Honestly Significant Difference) test: * = p<0.05. (PDF) [file pgen.1009162.s002.pdf]

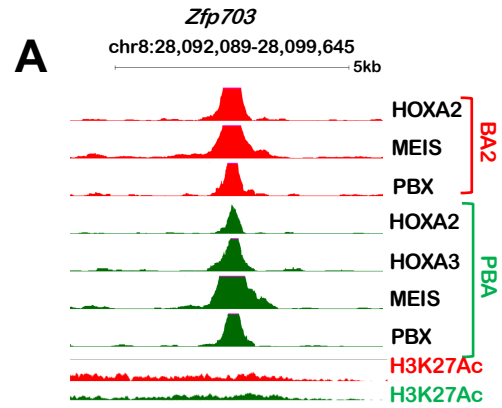

**C**

*Zfp703* wt: TTAGGACTGTCAGAGTGATTAAATGAGGG  
*Zfp703* mut: TTAGGACTGTCAGAGTGCCTCGTGAGGG

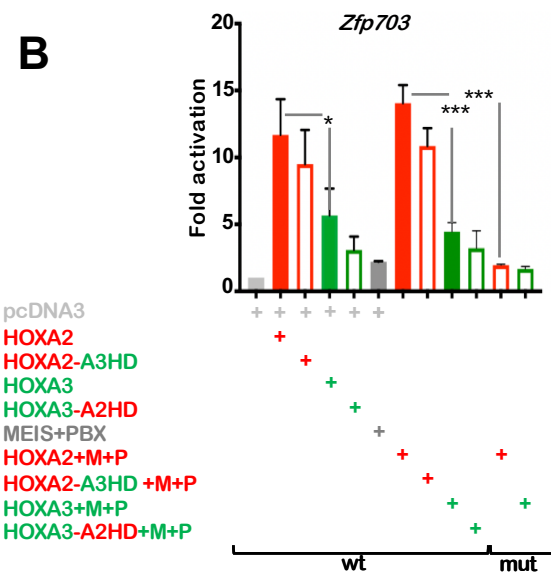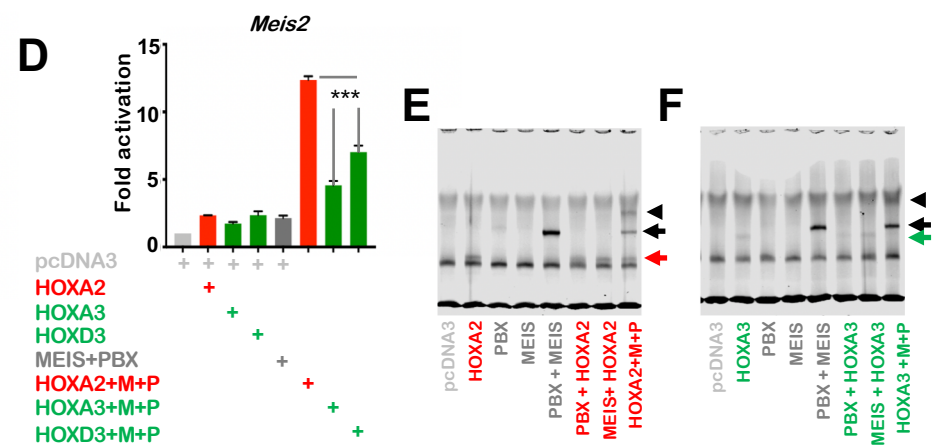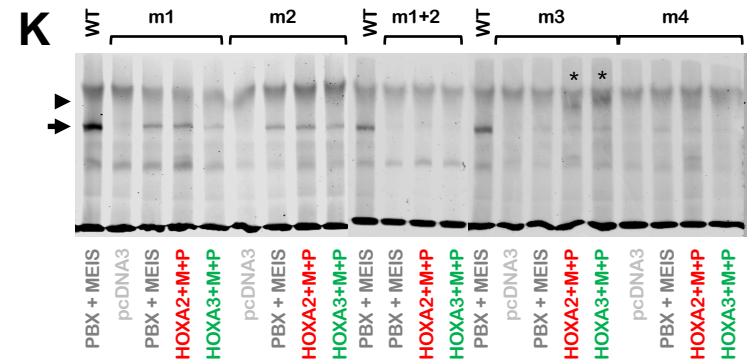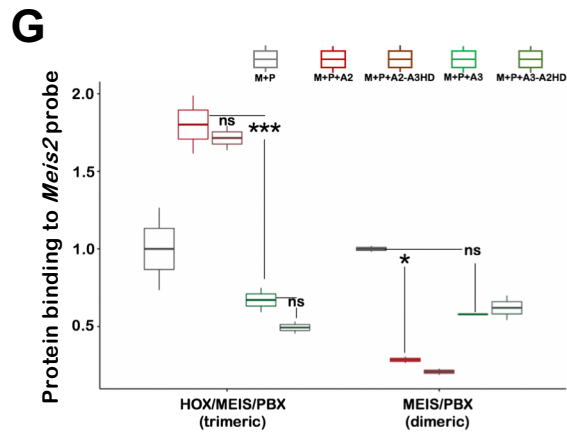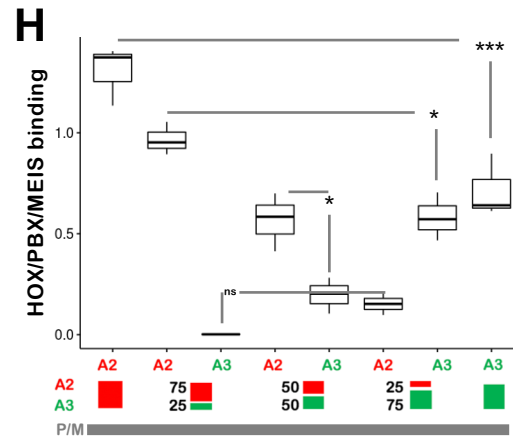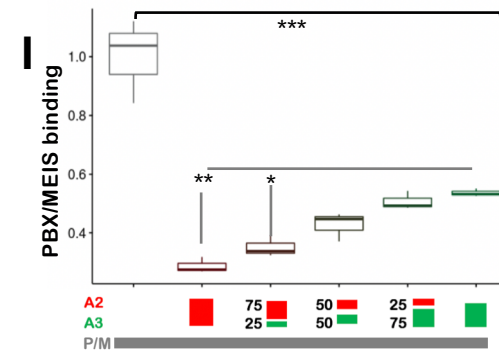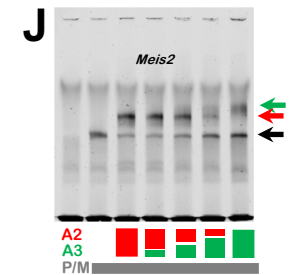

Supplement: S3 Fig — A. UCSC tracks of HOXA2, HOXA3, PBX, MEIS binding and H3K27 acetylation profiles in BA2 (red) and PBA (green) at the Zfp703 locus. Strong HOX and TALE binding is observed in both tissues, with higher acetylation levels in BA2. B. Luciferase activity driven by Zfp703 enhancer co-transfected with Hoxa2 (red bar), Hoxa3 (green bar), Hoxa2-a3HD (red empty bar), Hoxa3-a2HD (green empty bar), Meis2 and Pbx1a (grey bar) expression vectors, alone and in combination, in NIH3T3 cells. Addition of MEIS/PBX amplifies the difference in HOXA2 and HOXA3 activation abilities. Changing the HOX-PBX site (shown in C) reduces HOX-TALE activation to the levels observed with TALE alone (empty bars). Values represent fold activation over basal enhancer activity and are presented as the average of at least two independent experiments, each performed in triplicate. Error bars represent the SEM. One-way ANOVA with post-hoc Tukey HSD test: * = p<0.05; *** = p<0.0005. C. Sequence of Zfp703 wild-type and mutant enhancer encompassing HOX-PBX and MEIS sites. HOX-PBX and MEIS motifs are underlined. Nucleotide substitution in the HOX-PBX site are shown in red. D. Luciferase activity driven by Meis2 enhancer co-transfected with Hoxa2 (red bar), Hoxa3 (green bar), Hoxd3 (green bar), Meis2 and Pbx1a, alone and in combination. When co-expressed with MEIS and PBX, HOXA2 shows higher activation capacity than HOX paralog 3. Statistical analysis was performed as in B. EF. HOXA2 (E, red arrow) and HOXA3 (F, green arrow) weakly bind the Meis2 probe. MEIS and PBX bind DNA together (black arrow). HOXA2 (E) or HOXA3 (F) do not bind the Meis2 probe with either MEIS or PBX alone. Addition of HOXA2 and HOXA3 to MEIS/PBX results in a trimeric protein complex (arrowheads in E and F). G. Quantification of MEIS/PBX and HOX binding to Meis2 probe. Binding of MEIS/PBX (dimeric) and MEIS/PBX/HOX (trimeric) complexes is expressed as relative percentage to PBX/MEIS binding to Meis2 probe in the absence of any HOX. Dimeric [file pgen.1009162.s003.pdf]

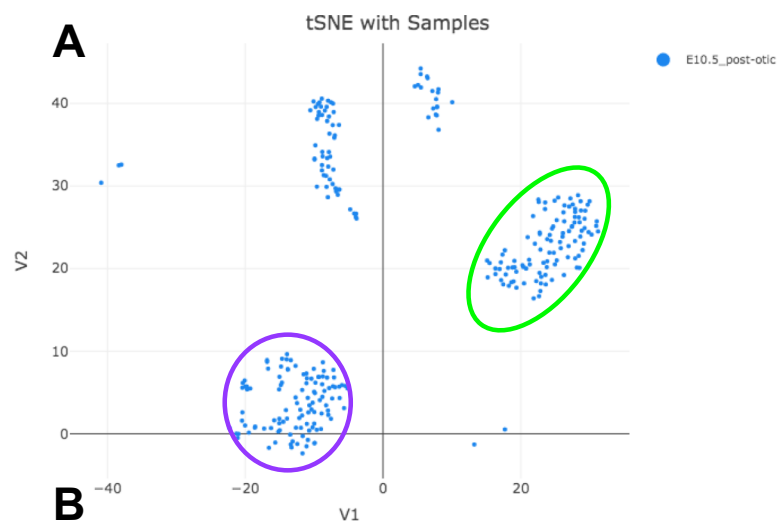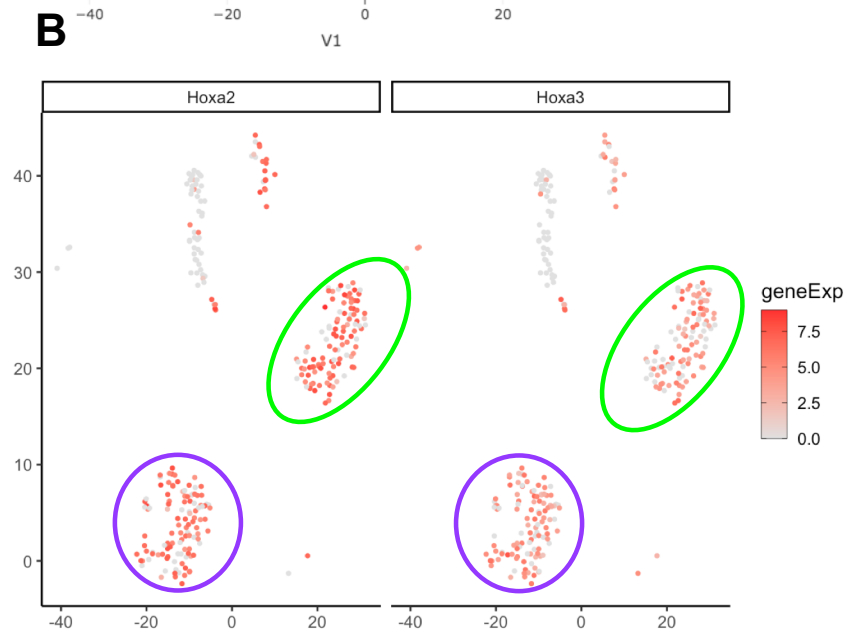

**C**

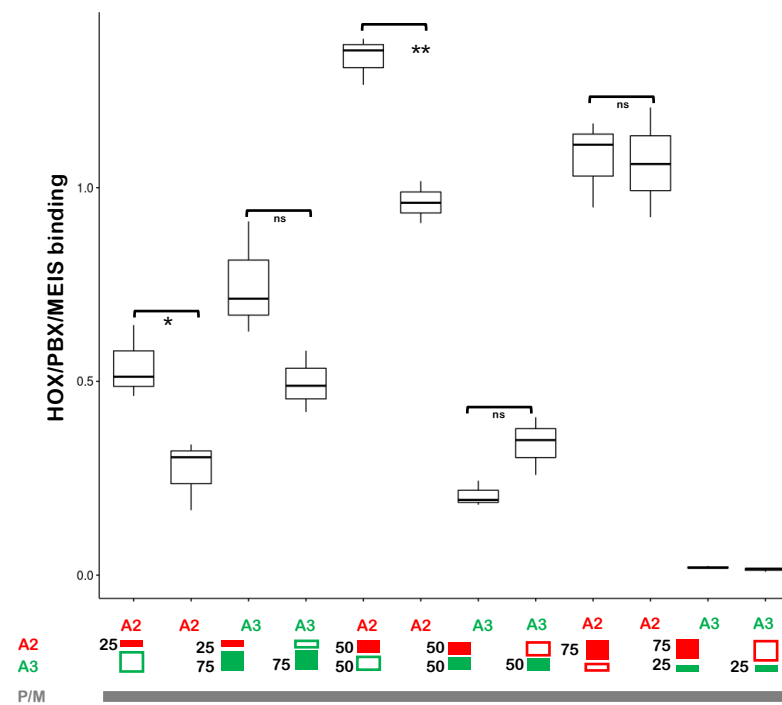

|         | Hoxa2 + | Hoxa2 - |
|---------|---------|---------|
| Hoxa3 + | 61*     | 10      |
| Hoxa3 - | 23      | 12      |
| P=0.017 |         |         |

|         | Hoxa2 + | Hoxa2 - |
|---------|---------|---------|
| Hoxa3 + | 56*     | 12      |
| Hoxa3 - | 12      | 9       |
| P=0.021 |         |         |

Supplement: S4 Fig — A. tSNE (T-distributed Stochastic Neighbour Embedding) of E10.5 post-otic cranial neural crest single cell RNA-seq (Soldatov et al. 2019) clusters cells into four main clusters. B. Two clusters (green and purple ovals in AB) exhibit high-expression of Hoxa2 and Hoxa3. Count of cell expressing Hoxa2 and Hoxa3 in the green and purple clusters shows that the majority of cells co-express Hoxa2 and Hoxa3, more than what is expected by chance (Fisher exact test). C. Quantification of HOX/MEIS/PBX binding to Meis2 probe shown in Fig 4E. Each HOX is co-translated with PBX/MEIS alone or with the other HOX. The highest HOX dose corresponds to 100%; HOXA2 (red) is translated at progressively decreasing levels, either alone, or co-translated with progressively increasing levels of HOXA3 (green) as indicated on the x-axis. At 50% and 25% levels, HOXA2 forms a significantly stronger trimeric complex (red arrow) on its own than when HOXA3 is added. (PDF) [file pgen.1009162.s004.pdf]

**A**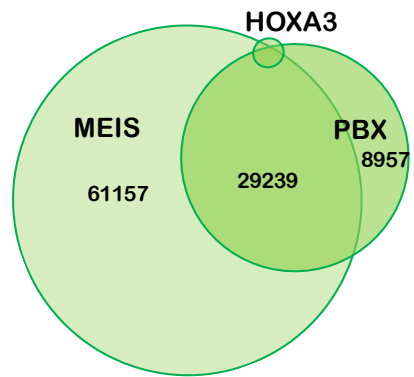**B**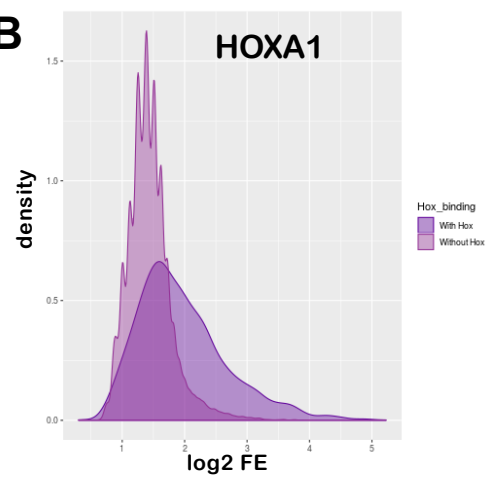**C**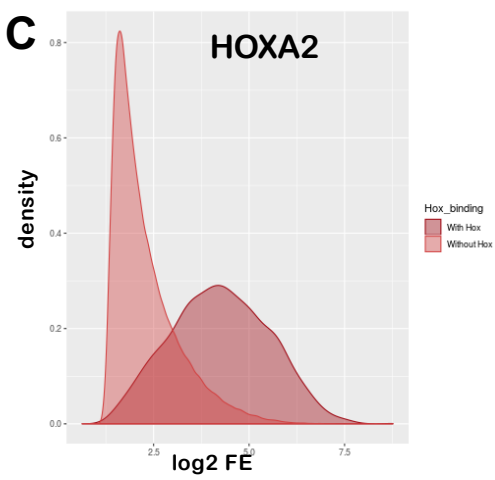**D**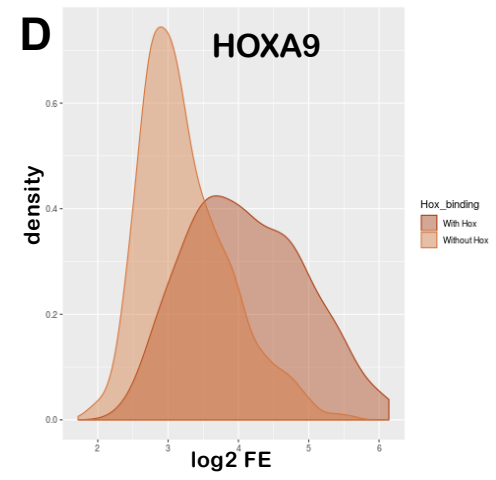

Supplement: S5 Fig — A. Whole Venn diagram of HOXA3, MEIS and PBX binding overlap, cropped in Fig 5A. Most MEIS and PBX peaks do not overlap with any HOXA3 peaks. B-D. Kernel density plots of MEIS peaks relative to FE. MEIS binding is sorted into peaks not overlapping HOX (lighter colour) and peaks overlapping HOX as indicated (darker colour). HOX and MEIS ChIP-seq in mouse embryonic stem cells (HOXA1), BA2 (HOXA2) and hematopoietic stem cells (HOXA9) were used in this analysis. (PDF) [file pgen.1009162.s005.pdf]

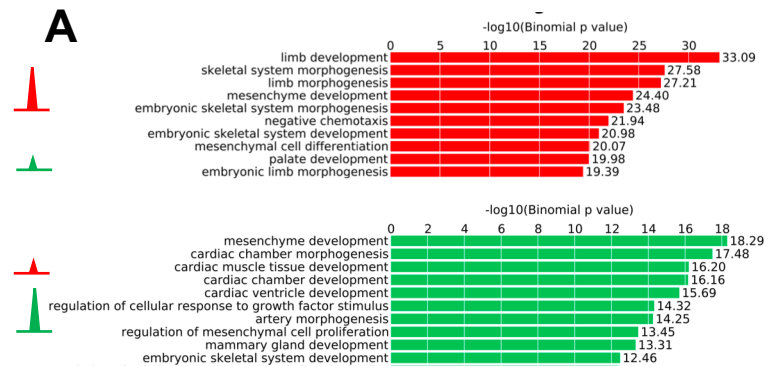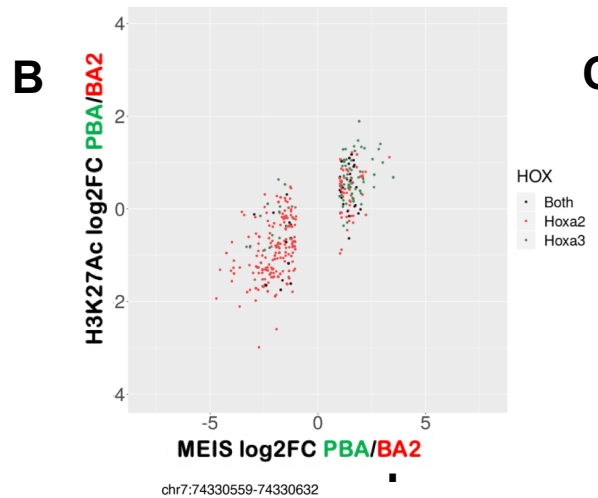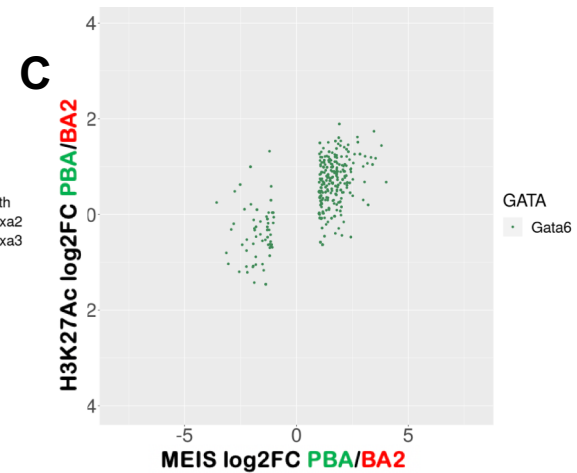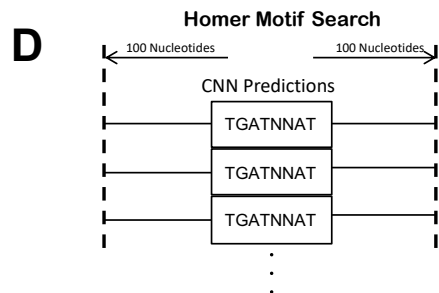

**E**

| TF family | de novo motif |
|-----------|---------------|
| Hox       | ATGATTATGAC   |
| Hox-Nkx   | CTAATG        |
| Meis      | CGCTGACAG     |
| Forkhead  | AAAATAAACG    |

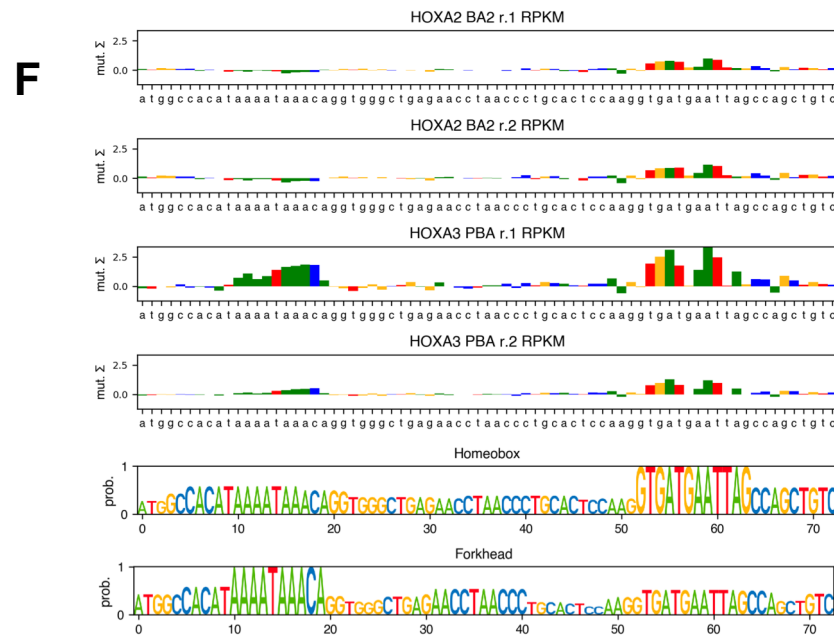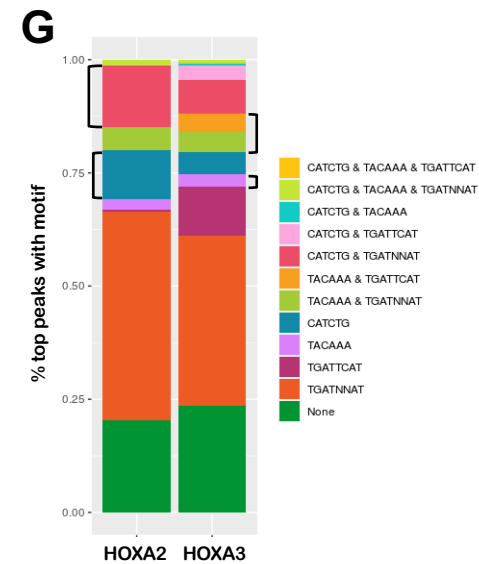

Supplement: S6 Fig — A. GREAT analysis of MEIS peaks higher in BA2 (relative to PBA, red) and PBA (relative to BA2, green). Fold change ≥5 across BAs is used as a cutoff. Differential MEIS peaks are associated with genes involved in different biological processes (top 10 categories are shown). BC. Fold change (PBA versus BA2) in MEIS binding and H3K27ac levels at HOX and GATA peaks. B. HOXA3 binding in PBA (green) and HOXA2 binding in BA2 (red) is associated with increased MEIS binding and H3K27ac in the corresponding tissues. C. Similarly, GATA6 binding in PBA is associated with increased MEIS and H3K27ac in PBA. DE. HOMER motif search in the vicinity of HOX/PBX sequence features identified by CNN (+/-100 nt around TGATNNAT, as shown in D) identifies enrichment of Forkhead motifs in MEIS higher PBA. Other enriched flanking motifs include MEIS and HD motifs. F. Example of HOX and Forkhead features, identified by CNN in a HOX- bound region. The RPKM feature tracks show combined RPKM change caused by mutating each nucleotide to its alternatives. Features of two experimental replicates for HOXA2 in BA2 and two replicates of HOXA3 in PBA are predicted by a jointly trained multitask model, trained by transfer learning from a MEIS RPKM model. Below, normalised cross-correlation of region sequence with HOX and Forkhead position weight matrix (PWM) obtained by k-mer counting is shown. Nucleotides in HOX and Forkhead motifs show increased sensitivity to perturbation, compared to their surrounding sequences. G. Motifs occurrence in top 250 HOXA2 and HOXA3 peaks (200 nt summits). Frequencies of single and co-occurring motifs are expressed as percentages and colour-coded in the bar plot. Square brackets indicate bHLH (CATCTG) in HOXA2 and Forkhead (TACAAA) in HOXA3 peaks. Motif frequency is calculated using IUPAC letters, as indicated. (PDF) [file pgen.1009162.s006.pdf]

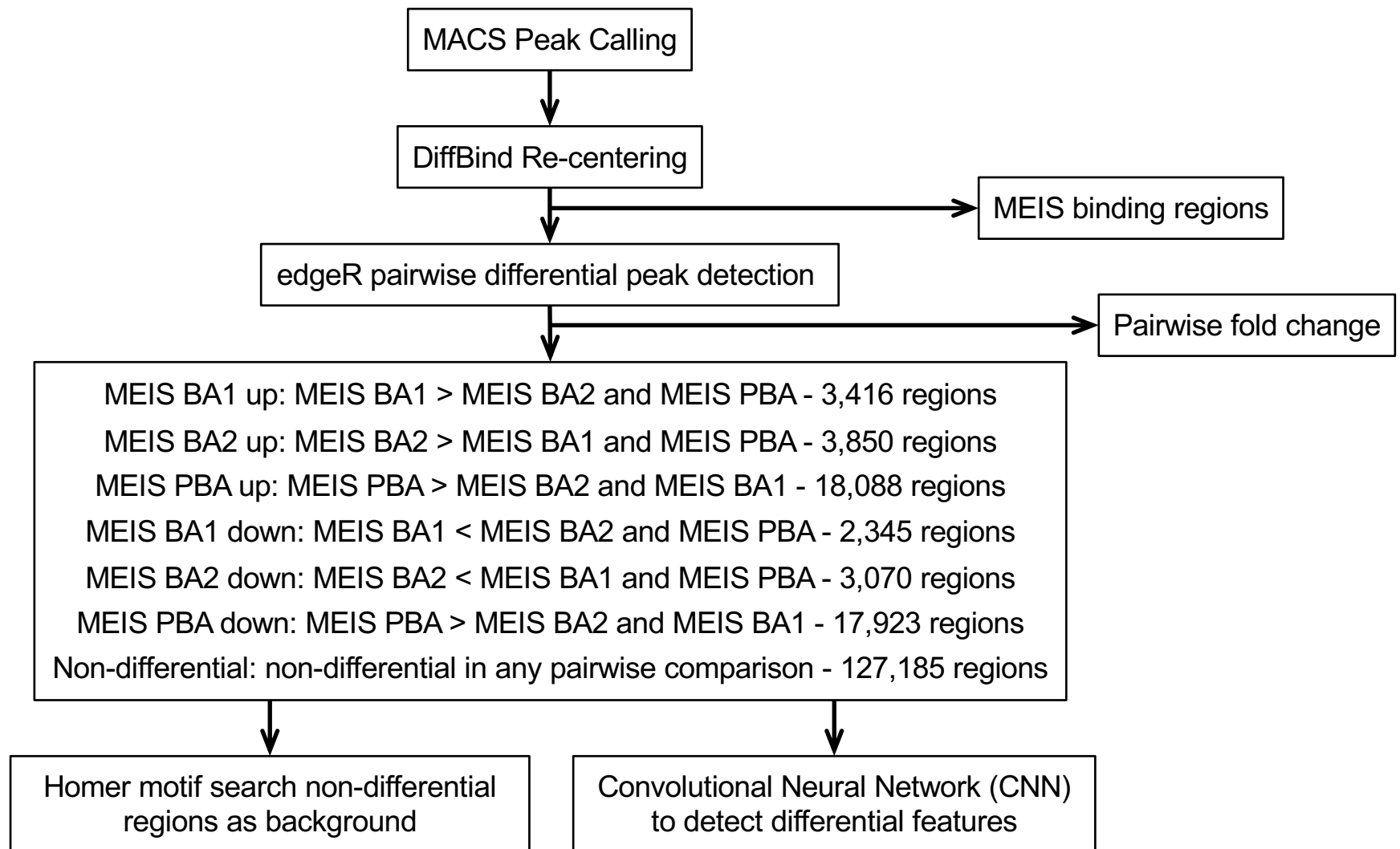

Supplement: S7 Fig — MACS peaks from MEIS ChIP-seq experiments in BA1, BA2 and PBA with replicates were recentered using DiffBind (see methods), and pairwise fold changes and differential binding regions were computed by edgeR as described (Phuycharoen et. al. 2019). Pairwise differential regions were combined into MEIS BA1 up, BA1 down, BA2 up, BA2 down, PBA up, and PBA down regions. Differential sequence motifs were detected as described (Phuycharoen et. al. 2019). Differential H3K27ac regions and fold changes were computed in the same manner. (PDF) [file pgen.1009162.s007.pdf]
