# Supplementary material for: HOX paralogs selectively convert binding of ubiquitous transcription factors into tissue-specific patterns of enhancer activation
Source: PLoS Genet. 2020 Dec 14;16(12):e1009162. doi: 10.1371/journal.pgen.1009162 (PMC7769617; doi:10.1371/journal.pgen.1009162)
Supplement: S3 Table — (DOCX) [file pgen.1009162.s010.docx]

Primers list:

**ChIP q-PCR**

*Zfp503* F: TCCTGGTCTGTTTAATGTTTGCT

R: GCCATCATCTAAAGCACAGAGG

*Zfp703* F: GGCTCCGATGGCTGTAATAA

R: TGCATTTGCAAAGACGGCTA

*Itih4* F: GAGTCTGCTTGGCTTGAACC

R: AAGATTGGGCACTTTTTGGA

*F2rl1* F: GTA GGT ACT GGC TTC AGG TCC

R: TTT GCT GCA GCG GTT CAT CC

*Wnt5a* F: AAGACCTCCTTGCGATCTGA

R: AAACACCAGGGGCAATCATA

*Prickle* F: TGC TCC AGG CAG CTA CTT CT

R: GAG TCC TTT CGT GGT AAA GGG

**Enhancers**

*Meis2* F: TGCCTTCATGTGAAGCAGTTCT

R: ACAGACACACAAATAGTCTACAGGG

*Zfp703* F: GGCTCCGATGGCTGTAATAA

R: TGCATTTGCAAAGACGGCTA
